# Reversing Bladder Cancer Chemo-resistance through Blocking Cell Senescence by a Combination Therapy

**DOI:** 10.7150/thno.133484

**Published:** 2026-05-11

**Authors:** Yirui He, Zaixiang Fang, Jiapeng Zhang, Qiang Wei, Yunkun Li, Zhenyu Duan, Gang Xu, Tianhai Lin, Qiao Xiong, Qiyong Gong, Pin Tan, Kui Luo

**Affiliations:** 1 Department of Urology, Department of Radiology, Institution of Radiology and Medical Imaging, Huaxi MR Research Center (HMRRC), Department of Hematology, Frontiers Science Center for Disease-Related Molecular Network, National Clinical Research Center for Geriatrics, State Key Laboratory of Biotherapy, West China Hospital, Sichuan University, Chengdu 610041, China.; 2 Psychoradiology Key Laboratory of Sichuan Province, West China Hospital, Sichuan University, Research Unit of Psychoradiology, Chinese Academy of Medical Sciences, Chengdu 610041, China.; 3 Xiamen Key Lab of Psychoradiology and Neuromodulation, Department of Radiology, West China Xiamen Hospital of Sichuan University, Xiamen 361021, China.

**Keywords:** drug resistance, cell senescence, bladder cancer, combination therapy, stimulation-responsive nanomedicine

## Abstract

**Rationale:**

Chemoresistance severely limits therapeutic options for advanced bladder cancer. Histone deacetylases (HDACs) have been implicated in tumour progression and treatment resistance, yet their role in acquired chemoresistance remains incompletely defined. We investigated whether epigenetic modulation could restore chemotherapy sensitivity in drug-refractory bladder cancer.

**Methods:**

Expression of HDAC1-3 was analysed in tumour tissues and correlated with clinical outcomes and chemoresistance-associated transcriptional programmes. A co-delivery therapeutic strategy combining the pan-HDAC inhibitor belinostat (PXD101) with paclitaxel (PTX) was developed and evaluated in patient-derived cisplatin-resistant organoids and a platinum-refractory patient-derived xenograft (PDX) model. Functional assays and genetic perturbation experiments were performed to delineate the underlying mechanisms.

**Results:**

HDAC1-3 were upregulated in bladder cancer tissues and associated with adverse prognosis and chemoresistance signatures. Combined PXD101 and PTX treatment significantly suppressed tumour growth in organoids and PDX models, with improved tolerability compared with standard regimens. Mechanistically, PXD101 attenuated senescence-associated programmes and reduced CDKN1A/p21 expression, thereby restoring PTX-induced antimitotic activity. Genetic manipulation identified p21 as a molecular switch linking therapy-induced senescence to chemoresistance. Modulation of p21 influenced cell-cycle re-entry, senescence burden, and responsiveness to PTX.

**Conclusions:**

These findings define an HDAC-p21-senescence axis that sustains chemoresistance in bladder cancer and provide preclinical evidence supporting combined epigenetic and antimitotic therapy as a strategy to overcome acquired drug tolerance.

## Introduction

Resistance to chemotherapy remains one of insurmountable challenges in clinical oncology treatment. For muscle-invasive bladder cancer (MIBC), chemotherapy is a highly effective treatment modality. However, the majority of MIBC undergoing chemotherapy ultimately develop chemoresistance, leading to treatment failure 1-3. Multifaceted factors have been identified for developing chemo-resistance, and a couple of mechanisms have been proposed including drug detoxification/inactivation, cell death inhibition or apoptosis suppression, dysregulated drug metabolism, enhanced DNA repair, gene amplification and epigenetic modifications 4-6.

Epigenetic dysregulation, e.g., aberrant histone modification, has been discovered in different cancer types 7-9. In bladder cancer, histone deacetylases (HDACs) are abnormally expressed, and their overexpression is often associated with poor therapeutic treatment outcomes and the development of chemoresistant phenotypes 10-13. This result suggests that HDAC inhibition may become a strategy to restore drug sensitivity in bladder cancer. However, the treatment with HDAC inhibitors (HDACi) in bladder cancer as a single modality generally leads to low therapeutic indexes. Belinostat (PXD101), an HDACi, had shown distinct activity in hematologic malignancies, but it has yielded limited benefits in solid tumors 14.

In this study, we revealed that PXD101 mitigated chemoresistance in bladder cancer by modulating chemoresistance-associated pathways, particularly through the CDKN1A/p21 axis. In resistant tumor cells, p21-driven senescence reinforced the cancer cell cycle arrest and enhanced the resistance of cancer cells to chemotherapy, in contrast, suppression of p21 re-sensitized dormant cancer cells to cytotoxic chemotherapeutic agents 15-17. Building on this rationale, we developed a combination strategy by integrating PXD101 with paclitaxel (PTX) in a drug delivery system. Encouraging treatment outcomes of the combination therapy were seen in patient-derived organoids and xenograft models of platinum-refractory bladder cancer. Mechanistic investigations suggested that after PXD101 attenuated the senescence programs, PTX exerted its canonical antimitotic action. Functional assays confirmed that p21 was a principal mediator of this process, therefore, the HDAC-p21-senescence axis could be targeted to mitigate chemoresistance. In summary, the therapeutic strategy of combining epigenetic modulation with conventional chemotherapy was successful in enhancing treatment responsiveness in bladder cancer, supported by both mechanistic investigations and preclinical experimental findings.

## Methods

### Materials and synthesis methods

Materials, and synthesis and characterization methods of PGP and PXD101@PGP nanoparticles were provided in Supporting Information.

### PDX model construction and *in vivo* tumor treatment

We constructed a PDX model by transplanting the tumor tissue obtained from a clinical patient with MIBC after radical cystectomy. Low-passage PDX tumors (P1–P3) were used for subsequent transplantation. Upon reaching a tumor volume of around 100 mm^3^, the nude mice were divided into six groups subjected to different treatments (n = 4) including saline as a control, PTX, PXD101, PTX + PXD101, PGP, and PXD101@PGP. However, due to severe toxicity observed in the PTX+PXD101 group, this group was excluded from data analysis. A therapeutic PTX dose of 7.0 mg kg^-1^ and a PXD101 dose of 24.5 mg kg^-1^ were applied to the groups treated with PGP and PXD101@PGP, respectively. The same PTX and PXD101 doses were also applied to the groups treated with a mixture or a combination of PGP and PXD101@PGP. They were injected intraperitoneally into the nude mice every 7 days. Two treatments within a total treatment cycle of 13 days were performed. Measurements of the width and length of subcutaneous tumors were conducted every 3 days with a vernier caliper. The tumor volume was calculated by the equation: V = LW^2^/2, where L and W were the length and width of the tumor, respectively. All nude mice were euthanized by cervical dislocation on the 13^th^ day. The subcutaneous tumor tissues and major organs of these nude mice were harvested. The wet weight of each isolated tumor tissue was measured. The harvested tissues were fixed using 4% paraformaldehyde (PFA), embedded, and subsequently stained by H&E and immunohistochemistry (IHC).

### Isolating and culturing bladder cancer primary tumor cells

We harvested fresh tumor tissues from the PDX nude mouse model. After cleaning and cutting these tumor tissues, they were kept in a 37 °C incubator for 1 h with a mixture of collagenase I + collagenase IV, and the digestion process was terminated by adding a DMEM medium containing 10% fetal bovine serum (FBS, Gibco, USA). The mixture was filtered using a 100 µM filter and centrifuged to obtain cell pellets. The cell pellets were resuspended in the DMEM complete medium and planted in a cell culture plate at an appropriate cell density (24-well plate: 5 × 10^4^ cells/well; and 6-well plate: 2 × 10^5^ cells/well). They were maintained at 37 °C with 5% CO_2_.

### *In vitro* cellular uptake and subcellular location

The digested tumor cells were planted on a coverglass bottom dish (size: 35 mm, density: 1 × 10^4^ cells/dish) and incubated for 48 h until they were adhered to the bottom and the cell shape was stretched. The spent medium was replaced with a DMEM medium without FBS containing a Hoechst 33342 nuclear dye (Thermo Scientific) and the cells were incubated for an additional hour. Cy5 or ^Cy5^PGP was added to the dish at an equivalent Cy5 concentration (1 µg mL^-1^) and the cells were kept in the incubator in the dark. At pre-specified time points, Hoechst 33342 signals for cell nuclei, Cy5 signals for intracellular PGP, and LysoTracker signals (Thermo Scientific) for intracellular lysosomes were observed in a high-power field of view under CLSM.

### Evaluation of cytotoxicity *in vitro*

A general cell culture procedure was applied for the following *in vitro* experiments. To evaluate *in vitro* cytotoxicity of PXD101@PGP, 3000 primary tumor cells per well were seeded into a 96-well plate and incubated for 48 h. After the cells were completely attached to a culture plate, the cell shape was observed to be stretched, and the cell confluence reached 70-80%, each drug formulation at different concentrations was added for another 24 h culture. Cytotoxicity of each formulation at a specific concentration was evaluated via a Cell Counting Kit-8 (CCK-8) assay.

### Apoptosis assay

After planting 2 × 10^5^ cells into each well of a 6-well plate, the cells were incubated for 48 h until they displayed a normal morphology. Different drug formulations at an equivalent PTX and PXD101 concentration of 2 and 7 µg mL^-1^, respectively, were added into the culture wells. After 24 h, the spent medium and non-attached cells were removed. According to the manufacturer’s instructions, AnnexinV and PI were added to label early apoptotic cells and late apoptotic/necrotic cells, respectively. 1 × 10^5^ cells were collected from each group to calculate the level of cell apoptosis after exposure to different formulations.

### Western blots

Tumor cells were prepared and treated by following a similar procedure for flow cytometry to detect apoptosis phenotypes. After removing the spent culture medium and washing the cells by PBS, 200 µL/well of a RIPA reagent was added to scrape and collect tumor cells. Proteins were extracted and diluted to the same concentration before complete electrophoresis in an SDS-polyacrylamide gel. After separation, the proteins were transferred onto a PVDF membrane (Invitrogen, USA) through an electrophoresis instrument and blocked with a 5% BSA solution. According to the instructions of the manufacturer, the electroporated PVDF membrane was sectioned and incubated overnight with primary antibodies diluted in a 5% BSA solution on a 4 °C shaker. After washing, the PVDF membrane was incubated with the corresponding secondary antibodies (diluted 1:10000) at room temperature for 1 h. After the PVDF membrane was washed with a TBST solution, an ECL solution was added dropwise before detection of the proteins.

### RNA-seq analysis

By following a similar procedure for apoptosis detection by flow cytometry, the primary tumor cells were obtained after 24 h culture. Using a Trizol reagent, the total RNA of tumor cells from each treatment group was extracted and stored at -80 °C. A standard Illumina protocol was used for the library construction for the RNA samples, and subsequent transcriptome-level sequencing was conducted on an Illumina NovaSeq 6000 platform.

To pre-process the Fastq data obtained from sequencing, low-quality bases, adapter sequences, and sequences less than 20 base pairs (bp) in length were removed. The STAR alignment tool (ver 2.7.10b) was used to construct the genome index and perform read comparison. FeatureCounts was applied to quantify the expression of each sample.

Quantitative results of gene expression were standardized via DESeq2 in Rstudio (ver 22.0). Differential gene expression analysis between groups was performed using DESeq2. Principal component analysis (PCA) was conducted based on normalized expression data, and data visualization was performed using the ggplot2 package.

### SA-β-galactosidase staining

To evaluate the cell senescence phenotype, sterile slides were pre-seeded in a 24-well plate. After planting 5 × 10^4^ cells/well, the cells were maintained for 48 h until they were completely adhered. PTX, PXD101, PTX + PXD101, PGP, and PXD101@PGP at an equivalent concentration of PTX and PXD101 were added for 24 h incubation. After observing morphological changes of these cells under a microscope and removing the spent medium, the cells were gently rinsed with PBS. According to the manufacturer's protocol, a glycosidase staining solution (250 µL/well) was prepared and incubated with the cells at 37 °C overnight. The dye solution was discarded, and the slide was gently removed from each well plate and fixed onto a glass slide with a neutral mounting medium for imaging and observation.

### Statistics

Numerical data were shown as mean ± standard deviation (SD), and group comparison was performed using Student's t-test. Comparison among multiple groups was conducted through one-way analysis of variance (ANOVA). Statistical analyses were performed via SPSS Statistics 26, and data presentation was executed via GraphPad Prism 8. The number of biological replicates (n) and specific statistical tests used for each analysis were indicated in the corresponding figure legends. No significant difference between two groups, denoted as 'ns', was determined by a p-value of above 0.05; and the degree of significance was presented with asterisks based on the p-value: * for p< 0.05, ** for p< 0.01, and *** for p< 0.001.

### Ethics

All human samples used in this study were obtained with approval from the Biomedical Ethics Committee of West China Hospital, Sichuan University (Approval Number: 2020-330). Informed consent forms were signed by all patients before biopsy samples were taken (version date: 2020/4/3). All animal procedures were performed in accordance with the Guidelines for Care and Use of Laboratory Animals of China and approved by the Animal Ethics Committee of West China Hospital of Sichuan University, China (No. 20240313001).

### Role of funders

Funders had no role in the study design, data collection, data analyses, interpretation, or in the writing of the report.

## Results

### Upregulated HDACs in bladder cancer and their roles in disease progression and chemoresistance

**Figure 1:**
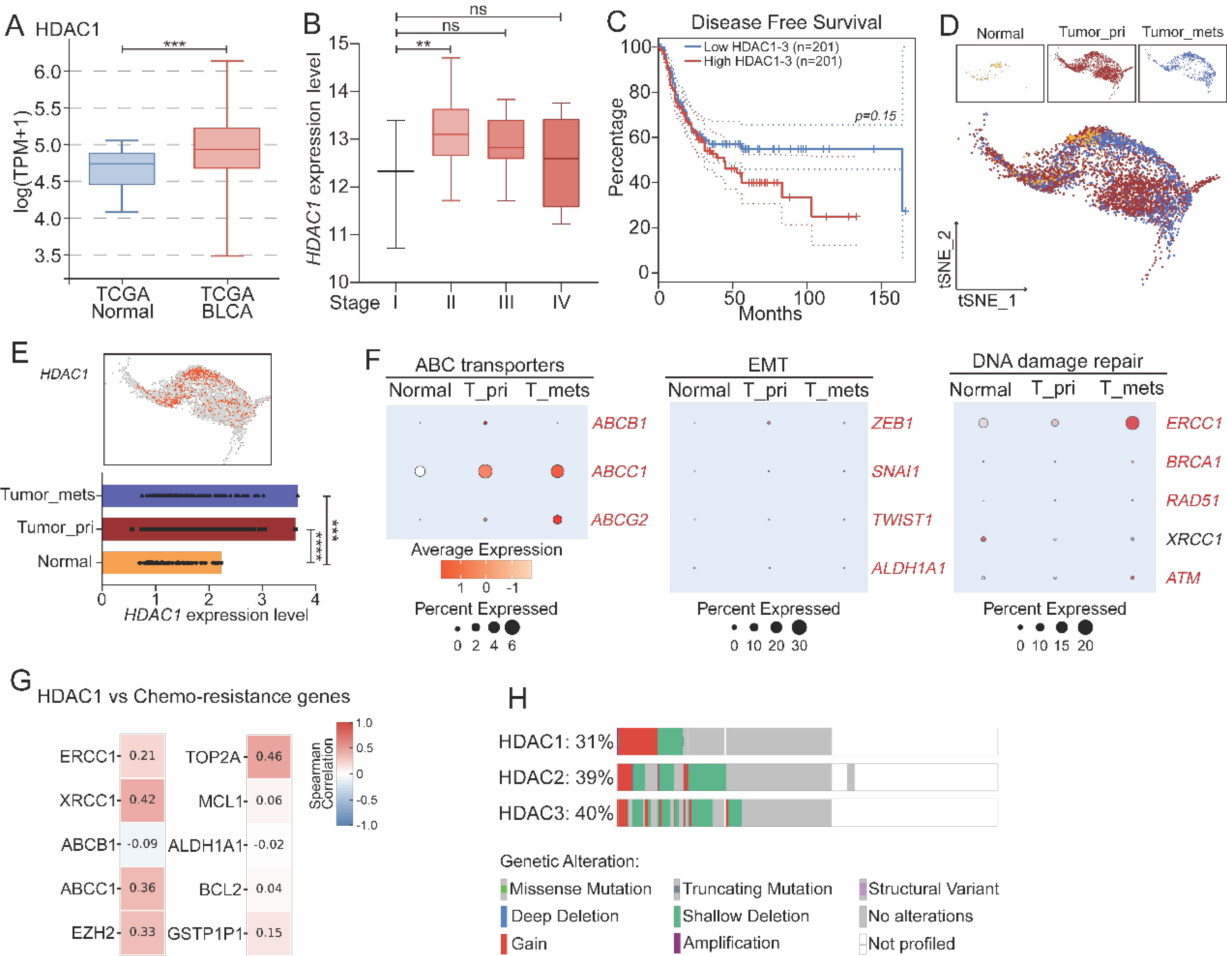
Clinical relevance and resistance-associated roles of HDACs in bladder cancer. (A) mRNA expression levels of HDAC1 in the TCGA-BLCA cohort. (B) Stage-specific expression levels of HDAC1 in bladder cancer. (C) Kaplan–Meier analysis of overall survival rates in TCGA-BLCA patients with low and high HDAC1-3 expression levels. (D-E) Relative expression of HDAC1 across epithelial cell types, based on scRNA-sequencing data. (F) Pathway enrichment analysis of the scRNA-seq data from epithelial tissues progressing from normal, primary (T-pri) and metastatic (T-mets) stages, and ABC transporters, EMT and DNA repair pathways in these tissues were distinguishably different. (G) Correlation of the HDAC1 expression level with the transcription level of representative resistance-associated genes (ABCC1, XRCC1, TOP2A, and EZH2) in the TCGA cohort. (H) Genomic alteration profiles of HDAC1-3. Predominant gained signatures of HDAC1-3 were found in bladder cancer.

To examine the clinical significance of the HDAC family member in bladder cancer, we studied the mRNA expression level of the HDAC family in the TCGA-BLCA cohort. Among all HDAC members, HDAC1 was the most consistently tumor-specific upregulated. The upregulation of HDACs displayed an increase with the progression from the early stage to the late stage, and the upregulation level was higher in T2-T4 tumors compared to that at T0-T1, although there was no statistical significance between two groups (Figure 1A-B; Figure S1-S2). We next assessed the prognostic relevance. Kaplan–Meier analysis indicated that a high expression level of HDAC1-3 was positively correlated with a poor overall survival rate (Figure 1C), suggesting the class I HDACs may have an impact on the therapeutic outcome. The HDAC1 level exhibited the most robust correlation with the overall survival rate, while HDAC2 and HDAC3 were moderately upregulated as the stage progressed (Figure S3), implying functional synergy within this subgroup.

To validate the variations in the expression level of class I HDACs at different tumor development stages, we performed scRNA-seq analysis of samples taken from normal urothelium, primary tumors, and metastatic lesions. HDAC1 and HDAC2 were predominantly expressed in malignant epithelial cells, but they were barely detected in the normal tissues (Figure 1D-E, Figure S4), suggesting they were specifically activated in the tumor tissues. Furthermore, along epithelial cell progression, our data showed progressive enrichment of chemoresistance-associated programs, including epithelial-mesenchymal transition (EMT), DNA repair, and ABC transporter pathways (Figure 1F; Figure S5). Correlation analysis revealed that the HDAC1 expression level was positively correlated with the transcriptional level of key resistance-related genes, such as ABCC1, XRCC1, TOP2A, and EZH2 (Figure 1G). In addition, patients with these enriched resistance genetic signatures had a significantly low survival rate in the TCGA cohort (Figure S6). However, stratification of these patients by the HDAC1 expression level did not completely recapitulate the low survival rate, suggesting that HDAC1 may play a role in a broad resistance-promoting network. Complementary to the transcriptional data, a gain in the copy number among HDAC genes, particularly HDAC1, was predominant among genetic variations, suggesting there may a widespread genetic variability of HDACs in tumor biology (Figure 1H).

### HDACi selection and cellular responses to PXD101-PTX co-treatment

**Figure 2:**
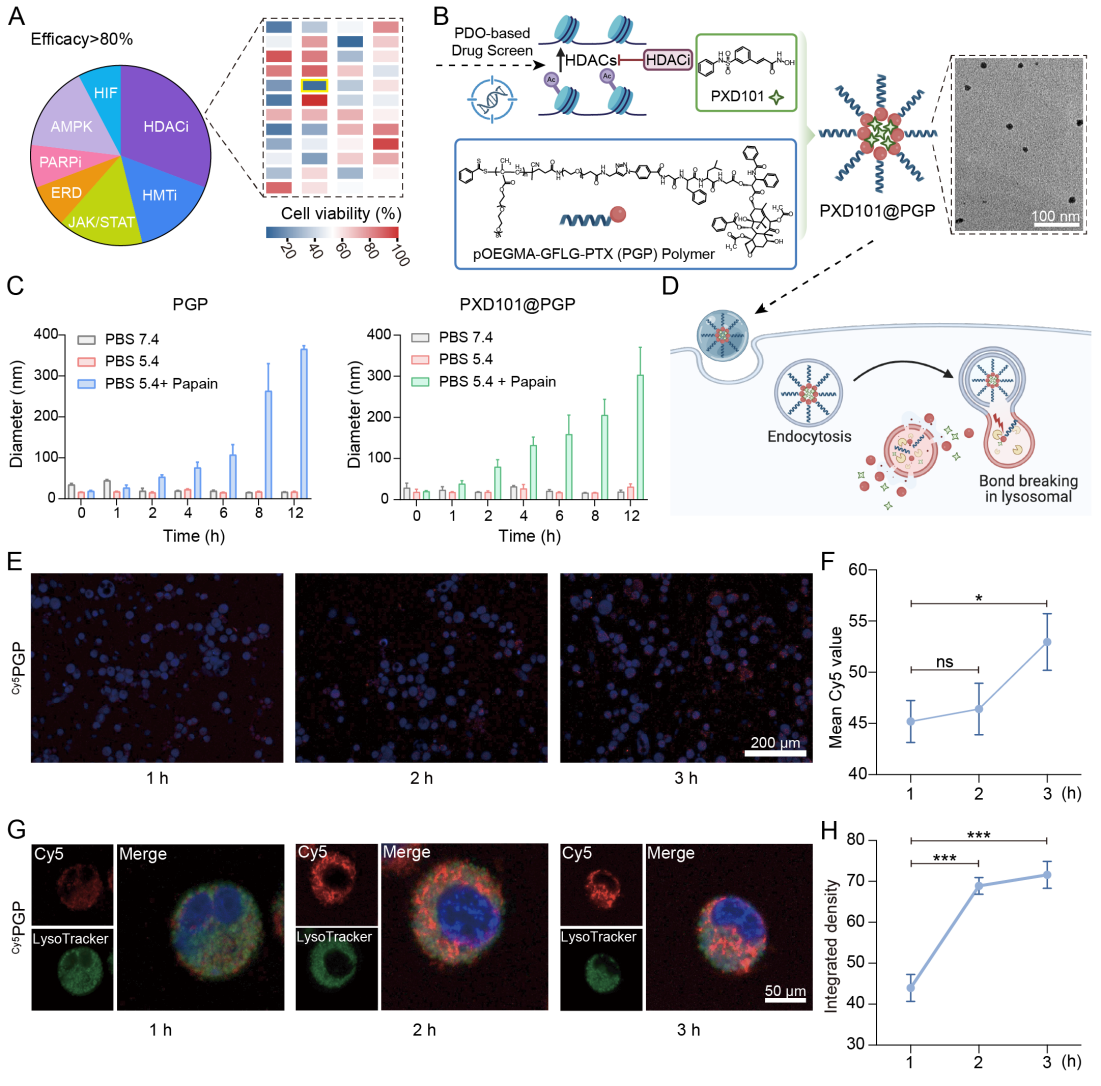
HDACi screening and characterization of the PXD101-PTX co-formulation. (A) Drug screening in cisplatin-resistant bladder cancer organoids. A superior efficacy was found from HDAC inhibitors, and belinostat (PXD101) was selected as the lead candidate. (B) Schematic illustration of patient-derived organoids (PDO)-guided selection of HDACi (PXD101) and its co-assembly with the pOEGMA-GFLG-PTX (PGP) polymer to form PXD101@PGP nanoparticles. A uniform spherical morphology of PXD101@PGP was observed in the TEM image (scale bar = 100 nm). (C) Size stability of PGP and PXD101@PGP under different buffer conditions including pH 7.4, pH 5.4, and pH 5.4 with papain that mimicked the condition in the endosome/lysosome. (D) Schematic illustration of the proposed intracellular itinerary of PXD101@PGP, from uptake to lysosomal degradation and drug release. (E) Representative CLSM images and quantification of the Cy5 signal intensity (F). Time-dependent uptake of Cy5-labeled PGP (^Cy5^PGP) was seen in primary bladder cancer cells. Hoechst 33342 (blue) for nuclei; Cy5 (red) for ^Cy5^PGP. Scale bar = 200 μm. (G) CLSM images for localization of ^Cy5^PGP in the endosome/lysosome at indicated time points. Hoechst 33342 (blue) for nuclei; LysoTracker (green) for endosomes/lysosomes. Scale bar = 50 μm. (H) Quantitative Pearson correlation coefficient (PCC) analysis for progressive colocalization between lysosomal signals and Cy5 signals from ^Cy5^PGP (mean ± SD, n=3, one-way ANOVA).

To evaluate the translational potential of HDAC inhibition, we screened a panel of epigenetic inhibitors by applying them in cisplatin-resistant bladder cancer patient-derived organoids. HDAC inhibitors (HDACi) emerged as the most potent drugs, and belinostat (PXD101) displayed the strongest inhibition efficacy, therefore, HDAC inhibition was selected for the combination with chemotherapy (Figure 2A).

To realize the combination therapy, we generated a PXD101-PTX co-delivery system (PXD101@PGP) by incorporating PXD101 into the pOEGMA-GFLG-PTX (PGP) polymer. The drug loading content (DLC) of PTX in PGP was 1.4% and the drug encapsulation efficiency (EE%) and DLC% of PXD101 in PXD101@PGP was 95% and 4.9%, respectively, (Figure S7-24). Transmission electron microscopy observations confirmed well-defined and uniform PXD101@PGP nanoparticles (Figure 2B, Figure S25). PGP and PXD101@PGP displayed considerable stability in PBS (pH = 7.4) and with a slight increase in the hydrodynamic diameter after exposure in DMEM with 10% fetal bovine serum (FBS), which indicated both PGP and PXD101@PGP were not adsorbed onto albumen during circulation in the body (Figure S26). To assess the responsiveness of PGP and PXD101@PGP, we examined the nanoformulation behavior under physiological and lysosomal-mimicking conditions. Both the PTX carrier and PXD101@PGP remained stable 5.4, whereas the presence of papain at pH 5.4 induced a marked increase in the hydrodynamic diameter of PXD101@PGP, confirming enzyme-triggered disassembly of PGP after cleavage of the GFLG segment in PGP by papain (Figure 2C). The drug release of PGP and PXD101@PGP were measured with HPLC. As shown in Figure S27, PTX exhibited negligible release in PBS (pH 5.4) for both PGP and PXD101@PGP, whereas the cumulative release approached 90% in the presence of papain. In addition, the cumulative release of PTX from PXD101@PGP was slightly slower than that from PGP, implying that the encapsulation of PXD101 may result in a more compact internal structure of the nanoparticles. Similarly, the cumulative release of PXD101 in PXD101@PGP remained stable at approximately 85% without enzyme conditions, while it increased to about 95% in the presence of papain.

A schematic in Figure 2D illustrates sequential processes of cellular uptake, endolysosomal trafficking, lysosome-mediated carrier disassembly and drug release of PXD101@PGP. To validate this process, we monitored intracellular uptake and trafficking. Confocal microscopy observations supported time-dependent internalization of Cy5-labeled PGP (named as ^Cy5^PGP, Figure S28) in primary chemotherapy-resistant tumor cells. The cytoplasmic fluorescence intensity increased from 1 to 3 h post-incubation (Figure 2E-F; free Cy5 as a control in Figure S29). The quantitative fluorescence signal intensity data confirmed a great level of ^Cy5^PGP accumulation within primary tumor cells.

Furthermore, co-localization assays using Hoechst and LysoTracker supported that ^Cy5^PGP progressively targeted lysosomes. After 1 h of incubation, Cy5 signals were diffused and partially overlapped with LysoTracker signals for lysosomes; at 2-3 h of incubation, the overlapping of the Cy5 signal with the lysosomal signal was markedly increased; and at 3 h the red Cy5 signal was almost completely overlapped with the green lysosomal signal (Figure 2G-H). The observations confirmed that ^Cy5^PGP entered endosomes and lysosomes after cellular uptake, verifying the proposed process in Figure 2D.

### *In vitro* anti-tumor effects of PXD101@PGP

**Figure 3:**
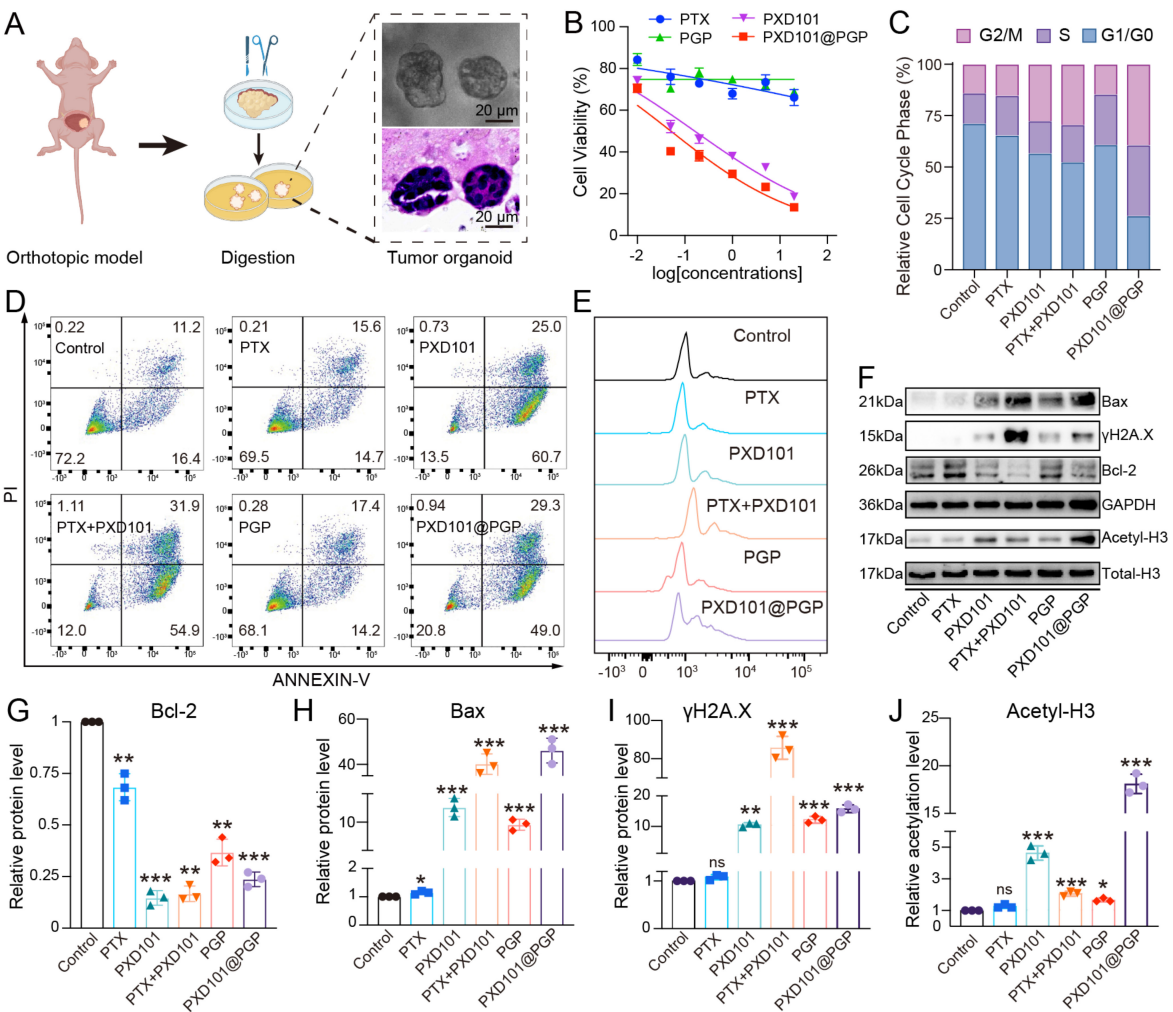
*In vitro* evaluation of drug responses in bladder cancer cells. (A) Schematic illustration of organoid construction from an orthotopic mouse model. (B) Cytotoxicity of different drug formulations at varying concentrations after 48 h treatment. (C) Cell cycle distribution and apoptosis analysis (D, E) of primary bladder cancer cells after 24 h treatment. (F) Western blotting of apoptosis-related proteins (Bax, γH2A.X, Bcl-2) and histone H3 acetylation after different treatments. (G–J) Quantification of the levels of Bax, γH2A.X, Bcl-2, and H3 acetylation from western blotting images (mean ± SD, n=3, one-way ANOVA).

To evaluate the synergistic cytotoxicity of PTX and PXD101 *in vitro*, we established platinum resistant bladder cancer organoids derived from an orthotopic tumor model (Figure 3A) 18. Cell viability assays revealed tumor cells displayed limited sensitivity to PTX alone, confirming a drug-resistant phenotype of these tumor cells. PXD101 monotherapy induced a concentration-dependent reduction in the viability of tumor cells, while the co-delivery formulation PXD101@PGP exerted the most pronounced cytotoxicity, indicating the synergistic activity of PXD101 and PGP (Figure 3B).

Cell cycle analysis revealed that PTX or PGP monotherapy displayed negligible changes in the cell cycle of tumor cells. PXD101 treatment resulted in a significant increase in the G2/M population. When PXD101 was combined with PTX, the cell cycle arrest at the G2/M phase was remarkably enhanced, and the strongest effect was observed in the PXD101@PGP group (Figure 3C). Apoptosis assays supported the cell cycle arrest results. PTX or PGP treatment induced negligible apoptotic distribution. Exposure to PXD101 resulted in a markedly elevated level of apoptosis. Encouragingly, both a mixture of PXD101 and PTX and PXD101@PGP induced distinct apoptosis and necrosis in the bladder cancer organoids, and they displayed a comparable efficacy (Figure 3D-E).

To investigate molecular regulation in the drug-resistant phenotype after treatment with PXD101@PGP and PXD101, we analyzed the proteins associated with apoptosis and chromatin. Western blot analyses revealed that PXD101, either alone or in combination of PTX (in the format of a physical mixture or PXD101@PGP), upregulated the level of acetylated histone H3, confirming effective HDAC inhibition. Meanwhile, pro-apoptotic Bax and the DNA damage marker γH2A.X were upregulated in the PXD101, PTX+PXD101, and PXD101@PGP groups (Figure 3F-J, Figure S30). These results suggested that PXD101 not only restored histone acetylation but also primed resistant tumor cells for PTX-induced apoptosis.

Collectively, these findings demonstrated that co-delivery of PTX and PXD101 synergistically reduced the cell viability, enhanced the cell cycle arrest at the G2/M phase and promoted apoptosis in chemotherapy-resistant bladder cancer organoids. Mechanistically, PXD101-mediated histone acetylation was found to alleviate resistance of primary chemotherapy-resistant tumor cells and invigorate their sensitivity to the cytotoxic effect of PTX.

### *In vivo* anti-tumor efficacy of PXD101@PGP

**Figure 4:**
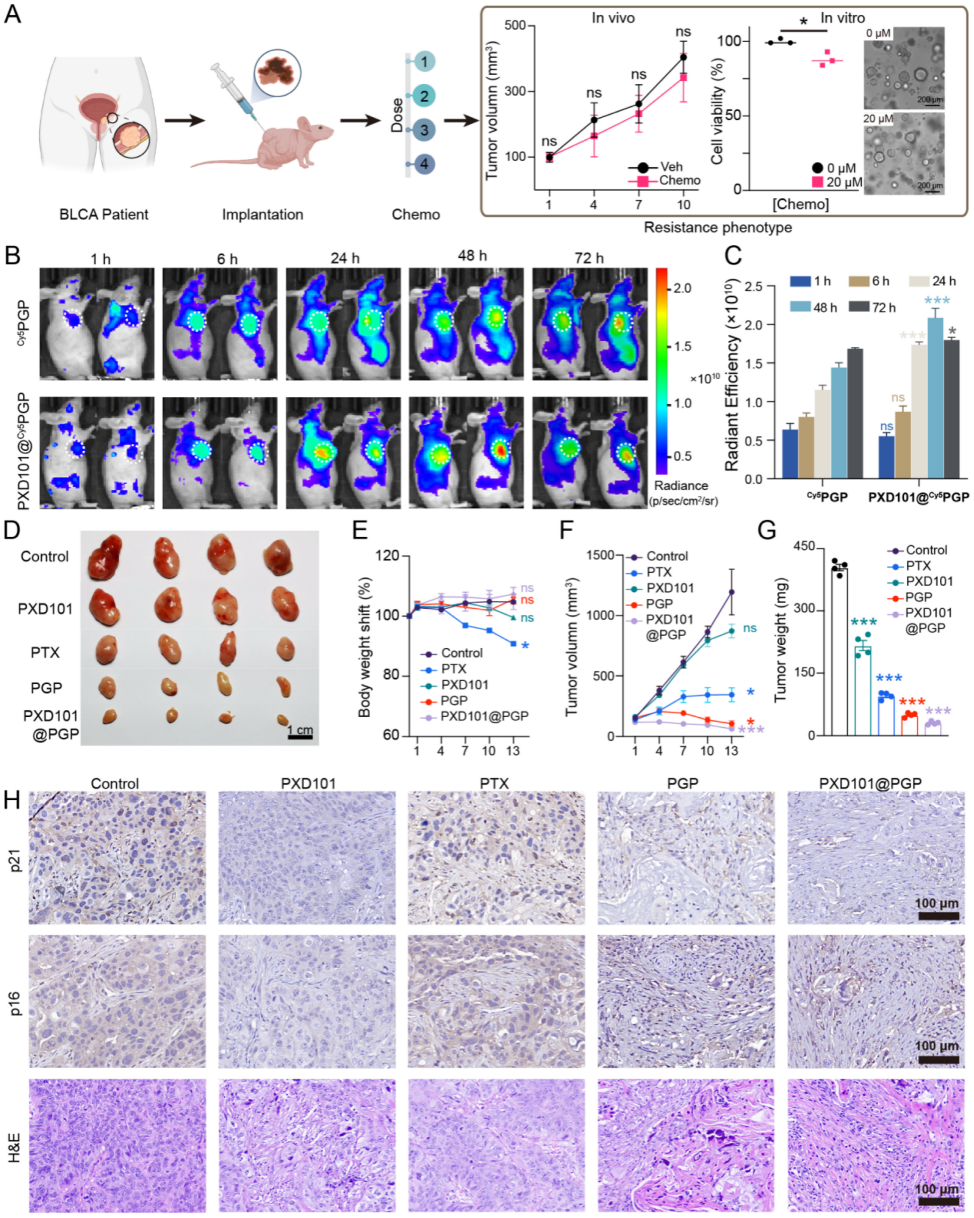
*In vivo* therapeutic efficacy of PGP and PXD101@PGP in a platinum-resistant bladder cancer PDX model. (A) Schematic illustration of the PDX model constructed from a platinum-refractory patient. Platinum-resistance was confirmed by the tumor size in the model after administration of PTX at a dose of 20 µM compared to the control group. (B) Representative images of ^Cy5^PGP and PXD101@^Cy5^PGP distribution *in vivo* at different time points after their intravenous injection (n = 4). (C) Average radiance efficiencies in subcutaneous tumors from the IVIS-imaged mice at different time points after ^Cy5^PGP or PXD101@^Cy5^PGP treatment (mean ± SD, n = 4 per group). Statistical significance was determined using unpaired two-tailed Student’s t-test at each time point. (D) Subcutaneous xenografts harvested from the mice at the end of the *in vivo* treatment regimen. Scale bar = 1 cm. (E) Body weight shifts, tumor volumes (F) and tumor weights (G) in the PDX mice after treatment with different formulations (mean ± SD, n = 4). Statistical significance was determined using one-way ANOVA. (H) Representative p21, p16, and H&E staining images of tumor tissues harvested from the mice receiving different treatments. Scale bar = 100 μm.

To evaluate the *in vivo* efficacy of PXD101@PGP against platinum resistant bladder cancer, we collected the tumor tissue from a patient with MIBC who had failed platinum-based chemotherapy and established a PDX model in nude mice. The transplanted tumors retained the clinically resistant phenotype, thus the PDX model was applied to evaluate therapeutic outcomes of PXD101@PGP (Figure 4A).

For biodistribution assessment, we employed Cy5-labeled PGP due to deep tissue penetration and low background signal of Cy5. Live animal images indicated that both ^Cy5^PGP and PXD101@^ Cy5^PGP accumulated at the tumor site, while the signal intensity of PXD101@^ Cy5^PGP was consistently higher than that of ^Cy5^PGP across the time points tested, indicating that the incorporation of PXD101 enhanced tumor retention of PGP (Figure 4B, C).

We next examined the therapeutic efficacy of PXD101@PGP. Tumor-bearing mice were randomized into six groups (n = 4 per group), including PTX, PXD101, PTX+PXD101, PGP, and PXD101@PGP; however, the PTX+PXD101 group was excluded from data analysis due to tolerability limitations observed during treatment. The remaining groups were administered intraperitoneally on days 0 and 7. Tumor growth was monitored every three days for 13 days. The tumor size in the saline group grew nearly 10 folds, and PXD101 monotherapy displayed a modest effect with an 8.7-fold increase in the tumor size. PTX treatment curbed tumor growth with a 3.4-fold expansion in the tumor size. Administration of PTX-containing PGP nanoparticles led to distinct tumor growth suppression, which may be due to improved tumor targeting by nanoparticles compared to PTX. Remarkably, treatment with PXD101@PGP not only effectively suppressed tumor growth but also efficiently shrank the tumor volume to the baseline size, and the shrinkage in the tumor volume was confirmed from the weight of the excised tumor mass (Figure 4D, 4F, 4G).

Systemic tolerability of PXD101@PGP was also evaluated. Mice treated with PTX experienced a significant loss in the body weight, indicating its strong toxicity *in vivo*. By contrast, animals receiving PXD101@PGP maintained a stable or even slightly increased body weight, which was comparable to that in the controls group, indicating that the co-delivery formulation pronouncedly mitigated systemic adverse effects (Figure 4E). In addition, histological examination of major organs revealed no observable tissue damage (Figure S31), suggesting PXD101@PGP had a favorable systemic safety profile.

Although free PXD101 exhibited potent cytotoxicity *in vitro*, its *in vivo* efficacy was markedly reduced, which may be due to insufficient intratumoral exposure at a threshold dose. The nanoformulation improved the pharmacokinetic window and sustained tumor retention of PXD101, thereby allowing more effective modulation of resistance-associated pathways and restoring PTX sensitivity in chemoresistant tumors.

Histological analyses of harvested tumor tissues were conducted to verify the therapeutic benefits of PXD101@PGP. H&E staining revealed extensive necrosis and fibrous remodeling in tumors from the group treated with PXD101@PGP. IHC staining supported that the expression levels of two senescence markers, p21 and p16, were elevated in the PTX-treated tumors, while their expression levels were markedly reduced after treatment with PXD101 or PXD101@PGP. Notably, in the PXD101@PGP group, the reduction in the staining intensity within tumor cells was evident although background signals were seen in the fibrotic area, suggesting that co-delivery of PXD101 and PTX effectively diminished the senescent phenotype of bladder cancer cells *in vivo* (Figure 4H).

### Mechanisms of action of PXD101@PGP

**Figure 5:**
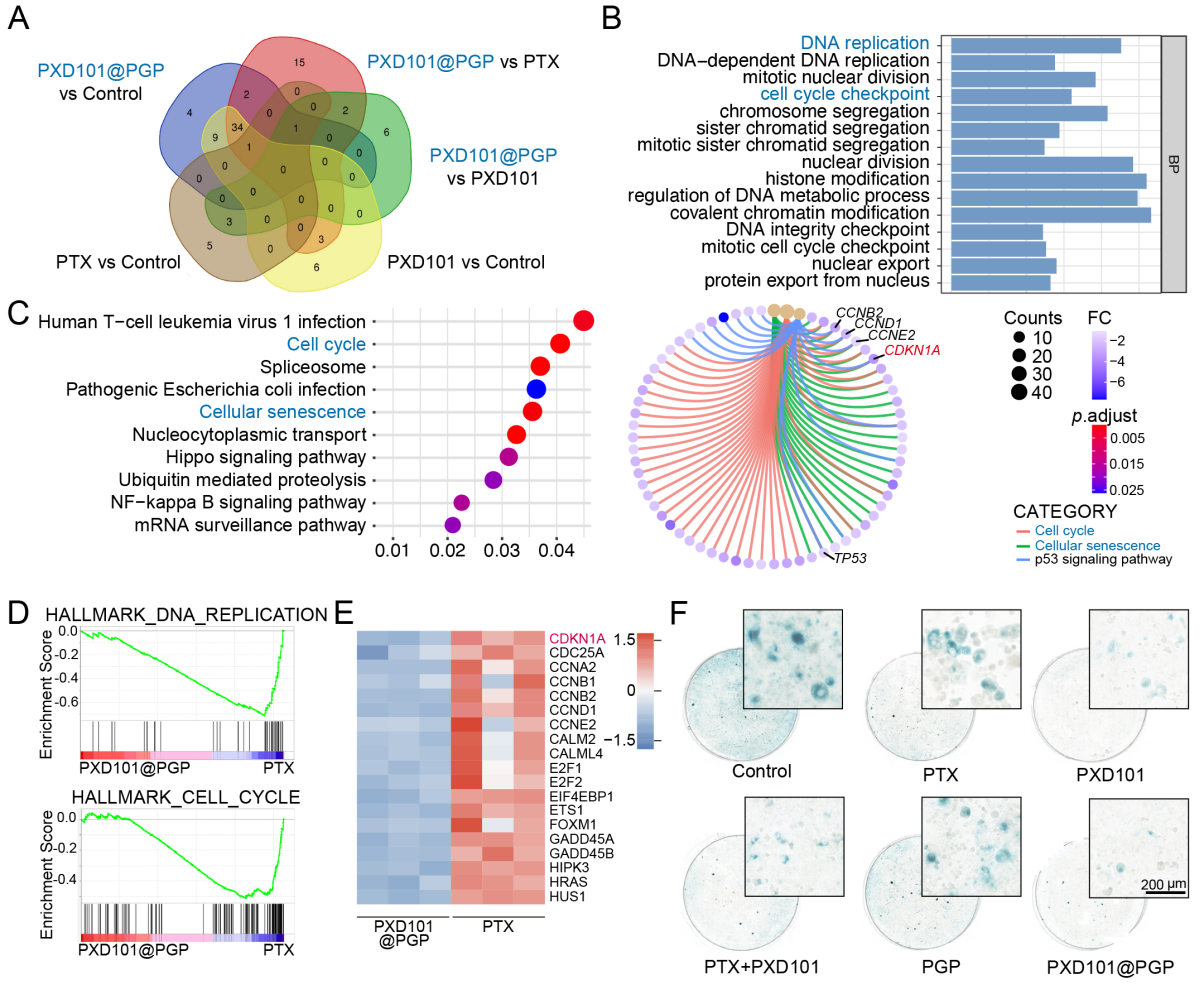
Mechanistic insights into the combination therapy of PTX and PXD101 in platinum-resistant bladder cancer. (A) Venn diagram for intersected enriched pathways from RNA-seq analysis of tumors under different treatments. (B) GO enrichment of up-regulated biological processes in the PXD101@PGP-treated group versus the PTX-treated group. (C) KEGG pathway enrichment and cnetplot visualization of up-regulated pathways in the PXD101@PGP group. (D) GSEA plots of the down-regulated DNA replication and cell cycle gene sets. (E) Heatmap of key differential genes in cell cycle and senescence pathways. (F) Representative SA-β-gal staining images of primary bladder cancer cells after 24 h treatment. Scale bar = 200 μm.

To elucidate molecular pathways involved in the therapeutic action of PXD101@PGP, we performed RNA sequencing to distinguish gene expression differences at the transcription level in *in vitro* chemo-resistant bladder cancer organoid samples after different treatments including PXD101, PXD101@PGP, PTX and the control. Comparison of differential gene enrichment between the group treated with monotherapy and the group treated with combination therapy suggested that the cellular senescence characteristic of bladder cancer cells could be diminished after exposure to PXD101 (Figure 5A-C). In addition, there was no statistical significance in the gene sets at the transcriptional level between the group treated with PTX and the control group, indicating that chemo-resistant tumor cells exhibited a reduced response to PTX monotherapy. However, the therapeutic effect of PTX was substantially enhanced in the group treated with PXD101@PGP because of the synergistic action of PXD101 and PGP. In the group subjected to the combination therapy, Gene Ontology (GO) term enrichment analysis of the cellular component (CC) indicated that PTX upregulated the MICROTUBE and CYTOPLASMIC MICROTUBULE pathways in tumor cells, suggesting the microtubule depolymerization inhibitory function of PTX was successfully restored through the inhibitory effect of PXD101 (Figure S32). Noticeably, the resensitization effect on the PTX cytotoxicity under the combination therapy regimen was pronounced.

Interestingly, Gene Set Enrichment Analysis (GSEA) of DEGs between the groups treated with PXD101@PGP and PTX suggested that PXD101 could suppress DNA REPLICATION and CELL CYCLE pathways in the group subjected to combination therapy (Figure 5D). These genetic characteristics may be associated with the regulation of cellular senescence by PXD101. Cellular senescence is characterized with sustained cell cycle arrest, while the optimal therapeutic effect of PTX can be achieved after it is applied to rapidly dividing cells. Therefore, cellular senescence in platinum resistant bladder cancer cells may diminish the anti-tumor function of PTX. Heatmaps analysis of DEGs revealed pronounced down-regulation of cell cycle-associated genes after the treatment with PXD101 via the co-delivery system (Figure 5E). Among these DEGs, CDKN1A (p21) was the most prominently downregulated, which was consistent with a decrease in the expression level of the senescence marker p21 in the PDX tumor model treated with PXD101 and PXD101@PGP via histological staining (Figure 4H). Meanwhile, SA-β-gal staining of *in vitro* tumor cells was performed to characterize the level of cellular senescence. The tumor cells after treatment with the formulations containing PXD101 exhibited a marked reduction in the level of cytoplasmic SA-β-gal (blue), supporting a decrease in the degree of cellular senescence (Figure 5F).

### p21-mediated chemosensitization through modulation of cellular senescence induced by PXD101@PGP

**Figure 6:**
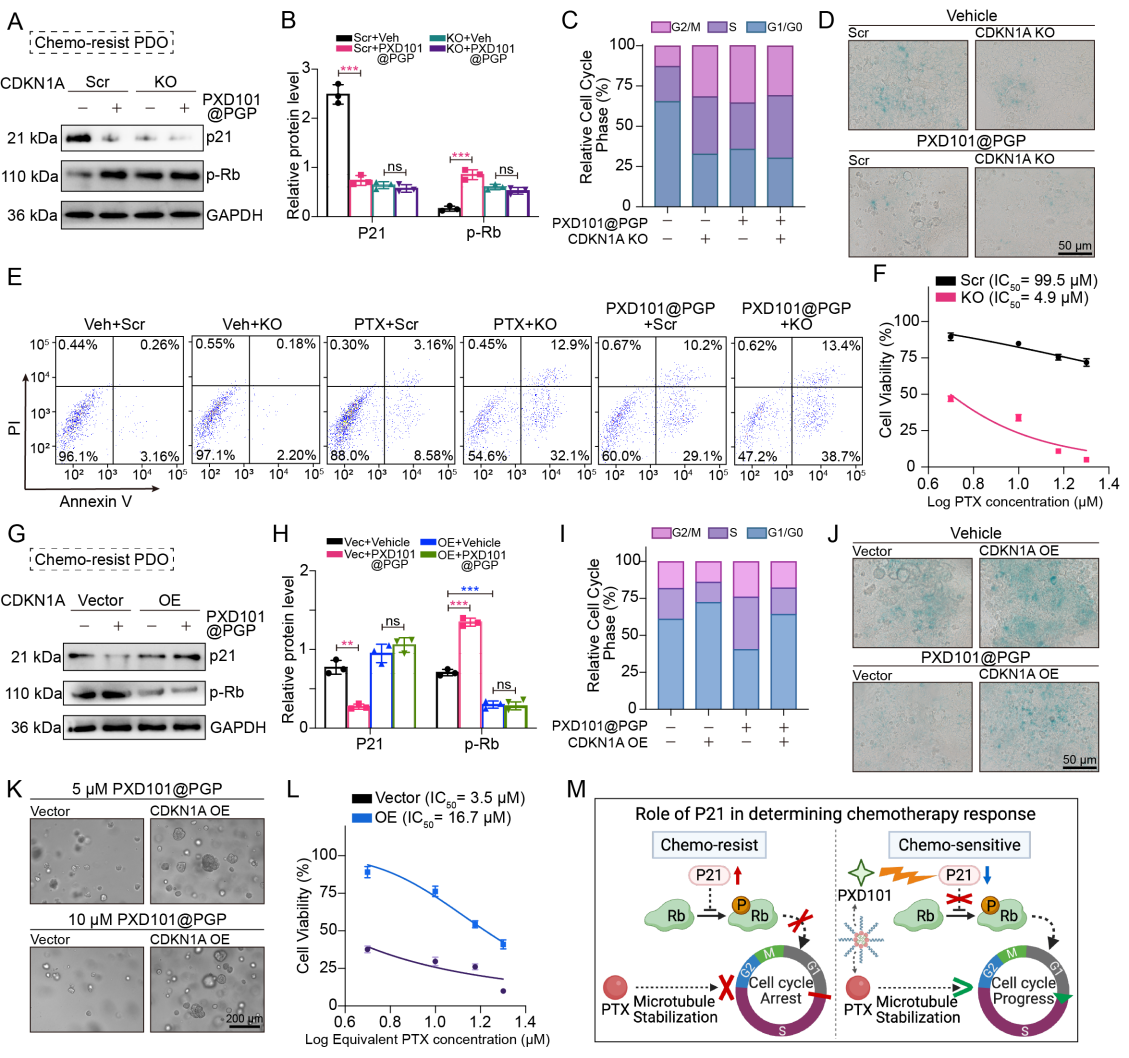
PXD101@PGP restored chemosensitivity by p21-mediated relief of cell-cycle arrest and reversal of senescence. (A-B) Effects of PXD101@PGP on p21 protein expression and downstream Rb phosphorylation in chemo-resistant tumor cells with or without CDKN1A knockout (KO) (mean ± SD, n=3, one-way ANOVA). (C) Cell cycle analyses and senescence staining images (D) in chemo-resistant CNKN1A KO organoids with or without PXD101@PGP treatment. (E) Apoptosis levels in chemo-resistant CDKN1A KO organoids treated with PTX or PXD101@PGP compared to the control groups treated with PTX or PXD101@PGP. (F) Restoration of the PTX sensitivity in CDKD1A KO organoids with a much lower IC50 value compared to the control group without KO. (G-H) Impact of PXD101@PGP on p21 expression and Rb phosphorylation in CNKN1A-overexpressing (OE) cells (mean ± SD, n=3, one-way ANOVA). (I) Cell cycle analyses and senescence staining images (J) of CDKN1A-OE organoids with or without PXD101@PGP treatment. (K-L) Enhanced resistance to PXD101@PGP in CDKN1A-OE organoids. (M) Scheme illustration of the role of p21 in modulating chemotherapy responses.

To determine the effect of p21 modulation on the therapeutic effects of PXD101@PGP, we examined the correlation between the p21 expression level and drug responses in patient-derived chemo-resistant bladder cancer organoids (PDOs). PXD101@PGP treatment markedly reduced p21 protein expression and enhanced Rb phosphorylation, indicating activation of cell cycle progression (Figure 6A-B).

We next assessed recapitulation of the effects of PXD101@PGP after p21 suppression. Notably, CDKN1A knockout (KO) phenocopied the effects of PXD101@PGP, which was evidenced by accelerated G1 phase progression and reduced cellular senescence. Importantly, marginal additional effects were observed when PXD101@PGP was applied to CDKN1A KO organoids, suggesting that p21 suppression may be a principal contributor to the drug-induced phenotype (Figure 6C-D). Consistently, CDKN1A KO restored chemosensitivity of organoids to PTX, which was supported by an elevated level of apoptosis and a markedly reduced IC_50_ compared to control organoids. Meanwhile, PXD101@PGP treatment induced a similar level of apoptosis in control organoids. However, no significant increase in the apoptosis level was observed when PXD101@PGP was applied to CDKN1A KO organoids, supporting that PXD101@PGP may exert its chemosensitizing effect predominantly through p21 suppression (Figure 6E-F).

To further reveal the role of p21 in PXD101@PGP-mediated therapeutic action, we generated CDKN1A-overexpressing (OE) organoids. In contrast to vector controls, CDKN1A overexpression markedly attenuated the effects of PXD101@PGP. Specifically, PXD101@PGP failed to effectively reduce p21 expression or promote Rb phosphorylation in CDKN1A-OE organoids. Correspondingly, persistent G1/G0 arrest and elevated senescence were maintained after PXD101@PGP treatment (Figure 6G-J). Furthermore, CDKN1A/p21 overexpression reduced the sensitivity of tumor organoids to PXD101@PGP, which was confirmed by increased tolerance to drug treatment and elevated PTX IC_50_ values compared to vector controls (Figure 6K-L). These findings indicate that enforced p21 expression may functionally counteract the effects of PXD101@PGP.

Taken together, these results support that p21 may act as a principal mediator for PXD101@PGP-induced chemosensitization by regulating cellular senescence and cell cycle progression, while additional HDAC-dependent mechanisms could play a role in PXD101@PGP-induced chemosensitization (Figure 6M).

## Discussion

Chemotherapy serves as a frontline therapy in the integrated management of bladder cancer, and it has shown promising therapeutic outcomes 19. Adjuvant or neoadjuvant chemotherapy has been demonstrated to eradicate potential micrometastases or metastatic lesions, thus prolonging the survival of patients. The emergence of innate or acquired chemotherapy resistance presents a major clinical challenge, narrowing therapeutic options for patients. Therefore, expanding the therapeutic window and identifying rational combination therapies to resensitize tumors to chemotherapy are of pressing clinical relevance 20, 21.

In a clinical setting, once resistance to platinum-based chemotherapy develops, subsequent treatment typically relies on non-platinum agents. Taxanes have served as common second-line or salvage therapeutic options 22. In this study, we observe that patient-derived bladder cancer cell organoids with primary resistance to cisplatin display diminished responses to PTX, suggesting these cells have developed downstream drug resistance through a variety of mechanisms. Importantly, a reduction in the drug responsiveness suggests that cells are in a state of partial drug tolerance rather than complete resistance, thereby providing a therapeutic window for pharmacological re-sensitization. Epigenetic alterations have been increasingly recognized as key contributors to non-genetic chemotherapy resistance in cancer cells 23. Among epigenetic alterations, histone deacetylation is frequently observed in MIBC and has been implicated in tumor progression and chemotherapy resistance 24. We confirm that HDACs, including HDAC1-3, are significantly overexpressed in bladder cancer tissues and the stage-specific overexpression level of HDACs is positively correlated with the poor survival rate of patients. Furthermore, single-cell transcriptomic profiling reveals HDACs are associated with chemotherapy resistance pathways and key resistance-related genes. These findings suggest that targeting HDACs may mechanistically restore drug sensitivity of bladder cancer cells and this approach can be translationally valuable.

To harness the findings, we proposed a therapeutic approach by combining pharmacological HDAC inhibition and disruption of the mitotic machinery to re-sensitize chemotherapy and enhance anti-tumor therapeutic effects for cisplatin-resistant bladder cancer. Despite promising benefits of pan-HDAC inhibitors (HDACi) in hematologic malignancies, their clinical application in certain solid tumors, such as transitional cell carcinoma (TCC), has been hampered because HDACi may induce non-specific epigenetic alterations, suboptimal pharmacokinetics and systemic toxicity 24. In this context, nanoparticle-based delivery systems have been demonstrated to improve tumor targeting and reduce systemic exposure 25-30. To address systemic toxicity associated with HDACi, we employed a pOEGMA-based nanocarrier to achieve tumor-targeting delivery of PXD101 as an HDACi and PTX as a microtubule-targeting anti-mitotic drug. This novel combination nanodrug, PXD101@PGP, displays favorable intracellular accumulation, improved cellular uptake, and lysosomal drug release (Figure 2) 31. In both *in vitro* and *in vivo* models, administration of PXD101@PGP significantly suppresses tumor growth, and the nanodrug formulation mitigates systemic toxicity, evidenced by no loss in the body weight and normal histology of major organs (Figure 4). The *in vivo* encouraging therapeutic effects of PXD101@PGP support the feasibility of co-delivery of therapeutic agents for epigenetic modulation and chemotherapy in a nanocarrier as a minimally toxic 32, highly effective intervention for treating cisplatin-resistant bladder cancer. It is noticed that there are minor discrepancies in the therapeutic efficacy derived from the measurements from the tumor volume and the tumor weight, and these discrepancies may be stemmed from inherent variabilities of caliper-based estimations compared with direct endpoint tumor weight measurements 33.

Encouragingly, the co-delivery system minimizes systemic toxicity of PTX, supporting the translational potential of PXD101@PGP in chemoresistant bladder cancer. The nanodrug formulation in a nanocarrier could also be extended to treat chemo-resistant cancer including breast cancer by incorporating an epigenetic regulator with a chemotherapeutic agent 18, 34, 35.

After confirming the synergistic effect of PTX and PXD101 in cisplatin-resistant bladder cancer, we delved into the mechanism of resensitizing bladder tumor cells to chemotherapy by PXD101@PGP. Transcriptomic analysis of bladder cancer cell samples under different treatment regimens reveals that PXD101@PGP can effectively downregulate cell cycle, DNA replication, and cellular senescence pathways. The inhibition of cellular senescence pathways is also achievable after PXD101 monotherapy, suggesting incorporation of PXD101 in the co-delivery system does not impact its biological activity. However, PXD101 monotherapy was insufficient to achieve robust cytotoxicity observed with the co-delivery system, indicating that modulation of senescence alone cannot account for the antitumor efficacy of PXD101@PGP.

To further dissect the mechanism, we compared transcriptomic profiles of tumors treated with PXD101@PGP and PXD101 monotherapy to identify PTX-induced signaling changes. This analysis reveals that PTX regains its anti-tubulin depolymerization function, which is evidenced by the upregulation of microtubule-associated cellular components within tumor cells in the combination regimen. These findings support that the antitumor effect of PXD101@PGP results from PXD101-mediated reduction of senescence-associated chemoresistance and restoration of the ability of PTX to block tubulin depolymerization.

There has been an ongoing debate on the impact of cellular senescence on tumor progression 36-38. Cellular senescence is increasingly recognized as a context-dependent process and it may be tumor-suppressive or tumor-supportive. While the induction of cell senescence may suppress tumor cell proliferation and restrain malignant progression 39, 40, accumulating evidence suggests that persistent or therapy-induced senescence may contribute to treatment tolerance and tumor recurrence, partially through sustained cell cycle arrest and the development of a senescence-associated secretory phenotype (SASP) 41. In this context, senescent tumor cells may evade cytotoxic therapies that preferentially target rapidly dividing cells 42, 43. It has been reported that cell cycle arrest in the G1 phase may be enhanced through p21, a CDK inhibitor, since it suppresses activity of several cyclin-CDK complexes. The aberrant overexpression of p21 has been implicated in the development of resistance to agents such as PTX 44. Our results support that, in chemoresistant bladder cancer, a p21-driven senescent state becomes a maladaptive cellular program that undermines chemotherapeutic action.

Directly targeting p21 remains challenging due to its dual functions and associated complex signaling pathways 45-47. Instead of direct targeting of p21, HDAC inhibitors could be employed to regulate p21 expression. It is noted that the regulation of the p21-driven senescent state via the CDKN1A/p21 axis by HDAC inhibitors is complex and biological context-dependent 48. HDAC inhibition has been widely reported to induce p21 expression through chromatin relaxation and transcriptional activation in many cellular systems, while suppression of p21 expression after HDAC inhibition has also been demonstrated by varying cellular states, treatment conditions, and epigenetic landscapes 49, 50. Importantly, HDAC inhibition does not indiscriminately eliminate all senescence programs, while it alleviates the resistance-associated senescent state.

We confirm p21 as a critical downstream effector of PXD101@PGP and regulation of p21 expression may be the mechanistic basis of chemosensitization. We consistently observe a reduction in p21 expression following PXD101 treatment (Figure 4H), supporting a transcription-associated downregulation of p21 in our experimental setting. This differential regulation may be due to cell state-specific transcriptional control or indirect regulatory mechanisms 48. In our model, downregulation of p21 is functionally correlated with attenuation of senescence-associated programs. Genetic ablation of p21 in the cancer cell organoids results in enhanced apoptosis, reduced senescence, and resensitization to PTX. The combination of genetic knockout of p21 with PTX achieved equivalent therapeutic effects as PXD101@PGP. Conversely, overexpression of p21 diminishes the sensitizing effect of PXD101@PGP. Persistent elevation in the p21 expression reinforces a dormant senescent phenotype, thereby diminishing the cytotoxic effects of PTX on inducing cell cycle arrest and DNA damage response (DDR). Therefore, p21 is a central checkpoint in the downstream of HDAC-dependent regulation, and it acts as a molecular switch between chemoresistant dormancy and chemosensitive proliferation. A reduction in p21 expression through PXD101 or its nanoformulation may facilitate cell cycle re-establishment and restore sensitivity of tumor cells to mitotic stress induced by PTX. Our *in vivo* experimental data support that PXD101 in combination with PTX can effectively suppress tumor growth and improve the survival rate in a PDX model. Therefore, functionally reprogramming senescence from a drug-tolerant state to a therapeutically targetable state may provide a conceptual framework for selectively targeting senescence-associated chemoresistance while preserving its context-dependent tumor-suppressive role.

It is noted that there are several limitations in the current study. Given the heterogeneity of chemoresistant cancer tissues, retrospective analyses (e.g., TCGA) could be conducted to comprehensively assess p21 as a biomarker. Although reproducible treatment responses have been demonstrated in the PDX animals, a relatively small cohort size (n = 4 per group) may weaken its statistical power, particularly in detecting modest treatment effects. A larger cohort will be employed in future studies to verify the findings from the proof-of-concept study. In addition, our current animal model could be refined to provide clinically relevant data. Specifically, a PDX model with high p21 expression could be established to allow *in vivo* validation of the efficacy of the combination therapy. A long-term culture model under continuous chemotherapeutic pressure could be employed to mimic clinical evolution of drug resistance and reveal p21-independent resistance mechanisms. These efforts will help elucidate a broader network underlying chemoresistance and provide new insights into precision oncology.

Overall, in this study, a mechanism-driven approach to targeting the HDAC-p21-senescence axis via PXD101 was combined with chemotherapy via PTX in a nanoplatform and the nanodrug formulation was evaluated in a clinically relevant PDX model of multidrug-resistant bladder cancer. HDAC inhibition results in suppression of p21 and resensitization of dormant tumor cells to therapeutic action of PTX, effectively addressing clinical challenges of chemoresistant bladder cancer.

## Supplementary Material

Supplementary materials and methods, figures.

## Abbreviations

MIBC: muscle-invasive bladder cancer
PTX: paclitaxel
PXD101: belinostat
PGP: pOEGMA-GFLG-PTX polymer
ABC: ATP-binding cassette
Cy5: cyanine 5
DEGs: differentially expressed genes
Rb: retinoblastoma protein
SA-β-gal: senescence-associated β-galactosidase
OE: overexpression
PDO: patient-derived organoids
PDX: patient-derived xenograft

## References

[1] Wéber A, Vignat J, Shah R, Morgan E, Laversanne M, Nagy P (2024). Global burden of bladder cancer mortality in 2020 and 2040 according to GLOBOCAN estimates. World J Urol.

[2] Parekh DJ, Reis IM, Castle EP, Gonzalgo ML, Woods ME, Svatek RS (2018). Robot-assisted radical cystectomy versus open radical cystectomy in patients with bladder cancer (RAZOR): an open-label, randomised, phase 3, non-inferiority trial. Lancet.

[3] Burdett S, Fisher DJ, Vale CL, Sternberg CN, Clarke NW, Parmar MK (2022). Adjuvant chemotherapy for muscle-invasive bladder cancer: a systematic review and meta-analysis of individual participant data from randomised controlled trials. Eur Urol.

[4] Wang Y, Wu X, Ren Z, Li Y, Zou W, Chen J (2023). Overcoming cancer chemotherapy resistance by the induction of ferroptosis. Drug Resist Updat.

[5] Salgado A, Zalba S, Lasarte JJ, Garrido MJ (2026). Emerging nanoparticle-based therapies for pancreatic cancer: Current clinical landscape. Adv Drug Deliv Rev.

[6] Ge M, Chen X-Y, Huang P, Fleishman JS, Yang D-H, Wu Z-X (2025). Understanding and overcoming multidrug resistance in cancer. Nat Rev Clin Oncol.

[7] Casadevall D, Kilian AY, Bellmunt J (2017). The prognostic role of epigenetic dysregulation in bladder cancer: A systematic review. Cancer Treat Rev.

[8] Mabe NW, Perry JA, Malone CF, Stegmaier K (2024). Pharmacological targeting of the cancer epigenome. Nat Cancer.

[9] Kiri S, Ryba T (2024). Cancer, metastasis, and the epigenome. Mol Cancer.

[10] Ellinger J, Schneider A-C, Bachmann A, Kristiansen G, Müller SC, Rogenhofer S (2016). Evaluation of global histone acetylation levels in bladder cancer patients. Anticancer Res.

[11] Plumb JA, Finn PW, Williams RJ, Bandara MJ, Romero MR, Watkins CJ (2003). Pharmacodynamic response and inhibition of growth of human tumor xenografts by the novel histone deacetylase inhibitor PXD101. Mol Cancer Ther.

[12] Shi M-Q, Xu Y, Fu X, Pan D-S, Lu X-P, Xiao Y (2024). Advances in targeting histone deacetylase for treatment of solid tumors. J Hematol Oncol.

[13] Nunes SP, Morales L, Rubio C, Munera-Maravilla E, Lodewijk I, Suárez-Cabrera C (2024). Modulation of tumor microenvironment by targeting histone acetylation in bladder cancer. Cell Death Discov.

[14] Hainsworth JD, Daugaard G, Lesimple T, Hübner G, Greco FA, Stahl MJ (2015). Paclitaxel/carboplatin with or without belinostat as empiric first-line treatment for patients with carcinoma of unknown primary site: A randomized, phase 2 trial. Cancer.

[15] Sharpless NE, Sherr CJ (2015). Forging a signature of *in vivo* senescence. Nat Rev Cancer.

[16] Wang C, Jiang X, Li H-Y, Hu J, Ji Q, Wang Q (2025). RIG-I-driven CDKN1A stabilization reinforces cellular senescence. Sci China Life Sci.

[17] Sturmlechner I, Zhang C, Sine CC, van Deursen E-J, Jeganathan KB, Hamada N (2021). p21 produces a bioactive secretome that places stressed cells under immunosurveillance. Science.

[18] Tan P, Cai H, Wei Q, Tang X, Zhang Q, Kopytynski M (2021). Enhanced chemo-photodynamic therapy of an enzyme-responsive prodrug in bladder cancer patient-derived xenograft models. Biomaterials.

[19] Li G, Song Z, Ru Y, Zhang J, Luo L, Yang W (2023). Small-molecule nanoprodrug with high drug loading and EGFR, PI3K/AKT dual-inhibiting properties for bladder cancer treatment. Explor (Beijing).

[20] Wang L, Jin C, Zhou J, Yin J, Xu G, Duan Z (2026). Nanomedicine in Organ Transplantation: From Graft Preservation and Repair to Immunomodulation and Monitoring. Theranostics.

[21] Li Y, Zhang X, Li Y, Sun L, Hu N, Lui S (2026). Neutrophil-based delivery platforms: from natural mechanisms to engineered therapeutics. Theranostics.

[22] Ko Y-J, Canil CM, Mukherjee SD, Winquist E, Elser C, Eisen A (2013). Nanoparticle albumin-bound paclitaxel for second-line treatment of metastatic urothelial carcinoma: a single group, multicentre, phase 2 study. Lancet Oncol.

[23] Karami Fath M, Azargoonjahromi A, Kiani A, Jalalifar F, Osati P, Akbari Oryani M (2022). The role of epigenetic modifications in drug resistance and treatment of breast cancer. Cell Mol Biol Lett.

[24] Li F, Zheng Z, Chen W, Li D, Zhang H, Zhu Y (2023). Regulation of cisplatin resistance in bladder cancer by epigenetic mechanisms. Drug Resist Updat.

[25] Blanco E, Shen H, Ferrari M (2015). Principles of nanoparticle design for overcoming biological barriers to drug delivery. Nat Biotechnol.

[26] Sun R, Xiang J, Zhou Q, Piao Y, Tang J, Shao S (2022). The tumor EPR effect for cancer drug delivery: Current status, limitations, and alternatives. Adv Drug Deliv Rev.

[27] Wang D, Qiu G, Wang H, Zheng L, Zhang X, Li Z (2025). Dual-Heterojunctions with Reversibly Photoactivated Structure Shift Alternately Decode Photocatalytic H2 Burst and Cascade Catalytic ROS Birth to Repress Cancer. Adv Mater.

[28] Zhu X, Li T, Wang Q, Yan K, Ma S, Lin Y (2024). Dual-Synergistic Nanomodulator Alleviates Exosomal PD-L1 Expression Enabling Exhausted Cytotoxic T Lymphocytes Rejuvenation for Potentiated iRFA-Treated Hepatocellular Carcinoma Immunotherapy. ACS Nano.

[29] Lei L, Song Y, Yang L, Wang Y, Xia X, Zhang Y (2025). Triethylamine-mediated protonation-deprotonation unlocks dual-drug self assembly to suppress breast cancer progression and metastasis. Proc Natl Acad Sci U S A.

[30] Lin L, Fang Z, Liu G, Liu Y, Li Z, Pan D (2025). Prodrug-based combinational nanomedicine remodels lipid metabolism for reinforced ferroptosis and immune activation. Acta Pharm Sin B.

[31] Li Y, Shen X, Ding H, Zhang Y, Pan D, Su L (2024). Dendritic nanomedicine enhances chemo-immunotherapy by disturbing metabolism of cancer-associated fibroblasts for deep penetration and activating function of immune cells. Acta Pharm Sin B.

[32] Li Y, Tong F, Wang Y, Wang J, Wu M, Li H (2024). *In situ* tumor vaccine with optimized nanoadjuvants and lymph node targeting capacity to treat ovarian cancer and metastases. Acta Pharm Sin B.

[33] Euhus DM, Hudd C, LaRegina MC, Johnson FE (1986). Tumor measurement in the nude mouse. J Surg Oncol.

[34] Zhang Y, Fang Z, Pan D, Li Y, Zhou J, Chen H (2024). Dendritic Polymer-Based Nanomedicines Remodel the Tumor Stroma: Improve Drug Penetration and Enhance Antitumor Immune Response. Adv Mater.

[35] Cheng X, Cai H, Li X, Zhang Y, Li S, Li Y (2026). Reversing adenosine-mediated immunosuppression in triple-negative breast cancer by synergistic chemo-immunotherapy via stimuli-responsive nanomedicines. EBioMedicine.

[36] Calcinotto A, Kohli J, Zagato E, Pellegrini L, Demaria M, Alimonti A (2019). Cellular senescence: aging, cancer, and injury. Physiol Rev.

[37] Schmitt CA, Wang B, Demaria M (2022). Senescence and cancer-role and therapeutic opportunities. Nat Rev Clin Oncol.

[38] Huang W, Hickson LJ, Eirin A, Kirkland JL, Lerman LO (2022). Cellular senescence: the good, the bad and the unknown. Nat Rev Nephrol.

[39] Faget DV, Ren Q, Stewart SA (2019). Unmasking senescence: context-dependent effects of SASP in cancer. Nat Rev Cancer.

[40] Ma L, Yu J, Fu Y, He X, Ge S, Jia R (2024). The dual role of cellular senescence in human tumor progression and therapy. MedComm.

[41] Francescangeli F, De Angelis ML, Rossi R, Cuccu A, Giuliani A, De Maria R (2023). Dormancy, stemness, and therapy resistance: interconnected players in cancer evolution. Cancer Metastasis Rev.

[42] Saleh T, Tyutyunyk-Massey L, Gewirtz DA (2019). Tumor cell escape from therapy-induced senescence as a model of disease recurrence after dormancy. Cancer Res.

[43] Loison I, Pioger A, Paget S, Metatla I, Vincent A, Abbadie C (2024). O-GlcNAcylation inhibition redirects the response of colon cancer cells to chemotherapy from senescence to apoptosis. Cell Death Dis.

[44] Zheng X, Yang E, Yan Y, Zhao S, Li X, Wang T (2025). AQB improves carboplatin sensitivity in endometrial cancer through dual DNA repair modulation: suppression of the p21-E2F1-RAD51 and ATF3-HDAC1-BRCA1 signaling. Cell Death Dis.

[45] Georgakilas AG, Martin OA, Bonner WM (2017). p21: a two-faced genome guardian. Trends Mol Med.

[46] Englund DA, Jolliffe A, Aversa Z, Zhang X, Sturmlechner I, Sakamoto AE (2023). p21 induces a senescence program and skeletal muscle dysfunction. Mol Metab.

[47] Tang Q, Tang K, Markby GR, Parys M, Phadwal K, MacRae VE (2025). Autophagy regulates cellular senescence by mediating the degradation of CDKN1A/p21 and CDKN2A/p16 through SQSTM1/p62-mediated selective autophagy in myxomatous mitral valve degeneration. Autophagy.

[48] Sachweh M, Drummond C, Higgins M, Campbell J, Lain S (2013). Incompatible effects of p53 and HDAC inhibition on p21 expression and cell cycle progression. Cell Death Dis.

[49] Pottier A, Park S, Lee Y, Liccardo F, Yang H, Park J (2025). TP53-agnostic lethality through combined pan-HDAC and CDK inhibition in acute myeloid leukemia. Cancer Lett.

[50] Wang L, Li H, Ren Y, Zou S, Fang W, Jiang X (2016). Targeting HDAC with a novel inhibitor effectively reverses paclitaxel resistance in non-small cell lung cancer via multiple mechanisms. Cell Death Dis.

