# Supplementary material for: Reversing Bladder Cancer Chemo-resistance through Blocking Cell Senescence by a Combination Therapy
Source: Theranostics. 2026 May 11;16(12):6827–42. doi: 10.7150/thno.133484 (PMC13232476; doi:10.7150/thno.133484)
Supplement: Supplementary file 1 — Supplementary materials and methods, figures. [file thnov16p6827s1.pdf]

Supporting Information

Reversing bladder cancer resistance through blocking cell senescence by a combination therapy

Yirui He,[a,1] Zaixiang Fang,[a,1] Jiapeng Zhang,[a,1] Qiang Wei,[a] Yunkun Li,[a] Zhenyu Duan,[a] Gang Xu,[a] Tianhai Lin,[a] Qiao Xiong,[a] Pin Tan,[a,*] Kui Luo [a,b,*]

[a] Department of Urology, Department of Radiology, Institution of Radiology and Medical Imaging, Huaxi MR Research Center (HMRRC), Department of Hematology, Frontiers Science Center for Disease-Related Molecular Network, National Clinical Research Center for Geriatrics, State Key Laboratory of Biotherapy, West China Hospital, Sichuan University, Chengdu 610041, China

[b] Functional and Molecular Imaging Key Laboratory of Sichuan Province, and Research Unit of Psychoradiology, Chinese Academy of Medical Sciences, Chengdu, 610041, China.

Email: uro_tanping@scu.edu.cn (Prof. Tan); luokui@scu.edu.cn (Prof. Luo)

[1] These authors made equal contributions to this work.

Contents

1. Experimental section 3

1.1 Materials and methods 3

1.2 Synthesis and Characterizations 4

1.2.1 Synthesis of N_3-GFLG-OH 4

1.2.2 Synthesis of N_3-GFLG-PTX 4

1.2.3 Synthesis of Fmoc-PEG_4-Alkynyl 5

1.2.4 Synthesis of CTA-PEG_4-Alkynyl 6

1.2.5 Synthesis of the POEGMA-Alkynyl polymer 7

1.2.6 Synthesis of the POEGMA-GFLG-PTX (PGP) polymer 7

1.3 GPC analysis of polymers 7

1.4 Drug loading content of PTX in PGP 8

1.5 Critical assembly concentration (CAC) of PGP 8

1.6 Preparation and characterization of PGP and PXD101@PGP 9

1.7 The responsiveness of PGP and PXD101@PGP 9

1.8 The stability of PGP and PXD101@PGP in DMEM with 10% FBS 10

2. Supplementary Results 11

2.1 Supplementary Schemes 11

2.2 Supplementary Figures 12

3. Reference 26

1. Experimental section

1.1 Materials and methods

Materials: Paclitaxel (PTX), belinostat (PXD101), propargulamine and 4-cyanopentanoic acid dithiobenzoate (CTA) were obtained from Macklin (Shanghai, China). Oligo (ethylene glycol) methyl ether methacrylate (OEGMA, M_W = 500) was purchased from Sigma-Aldrich. Cyanine5 azide (N_3-Cy5) and Fmoc-15-amino-4,7,10,13-tetraoxapentadecacanoicacid (Fmoc-PEG_4-COOH) were bought from Xi'an Confluore Biological Technology Co. Ltd (Xi'an, China). O-(1H-benzotriazolL-1-yl)-*N*, *N*, *N'*, *N'*- tetramethyluronium hexafluorophosphate (HBTU), 1-hydroxybenzotriazole (HOBt), and 2-(7-Azabenzotriazol-1-yl)-*N*, *N*, *N'*, *N'*-tetramethyluronium hexafluorophosphate (HATU) were obtained from GL Biochem Ltd. (Shanghai, China). *N*, *N*-diisopropylethylamine (DIPEA), 4-azidobenzoic acid, and 4, 4'-azobis (4-cyanovaleric acid) (ACVA) were obtained from Aladdin Reagent Inc. (Shanghai, China). Dimethylsulfoxide-d_6 (DMSO-d_6) was obtained from Energy Chemicals (Shanghai, China). *N*, *N*-dimethylformamide (DMF), dichloromethane (DCM), ethyl acetate (EA), methanol (MeOH), dimethyl sulfoxide (DMSO), and petroleum ether (PE) were bought from Chengdu Kelong Chemical Co. Ltd (Chengdu, China). TFA·NH_2-GFLG-OMe was synthesized via a previously reported method[1].

Methods:

^{1}H and ^{13}C NMR spectra were recorded on a Bruker Avance II NMR spectrometer at 400 MHz and 100 MHz. ^{1}H NMR (400 MHz) and ^{13}C NMR (100 MHz) chemical shifts were recorded relative to DMSO-d_6 as an internal reference (DMSO-d_6: δH = 2.50 ppm; δC = 39.52 ppm). Liquid chromatography-mass spectrometry (LC-MS) spectra were acquired via Agilent LC-MS 1260-6120. An inductively coupled plasma optical emission spectrometer (ICP-OES, Agilent) was used to measure the content of copper in PGP. Fourier-transform infrared (FTIR) spectra of N_3-GFLG-PTX and PGP were recorded on a Bruker INVENIO R IR spectrometer.

1.2 Synthesis and Characterizations

1.2.1 Synthesis of N_3-GFLG-OH

4-Azidobenzoic acid (1.772 g, 10.864 mmol, 1.1 eq), HBTU (5.618 g, 14.814 mmol, 1.5 eq) and HOBt (1.915 g, 14.814 mmol, 1.5 eq) were placed into a 250 mL flask which was on an ice bath. After they were dissolved in 100 mL DMF, 6.5 mL DIPEA (5.105 g, 39.504 mmol, 4.0 eq) was added into the flask. Finally, a DMF solution (50 mL) of TFA·NH_2-GFLG-OMe (4.969 g, 9.876 mmol, 1.0 eq) was added slowly and the reaction proceeded at room temperature and monitored by thin layer chromatography (TLC). After NH_2-GFLG-OMe was consumed, 300 mL EA and H_2O was added to separate the product through a separating funnel. The EA phase was washed with $NaHCO_3$ aq. (satd), 1 N HCl, and NaCl aq. (satd) sequentially. The organic solvent was removed by rotary evaporation after drying with Na_2SO_4. The residue was purified with column chromatography with a mobile phase of DCM/MeOH (20/1, v/v), and 3.831 g of a white solid was obtained at a yield of 70%.

N_3-GFLG-OMe (3.831 g, 6.938 mmol, 1.0 eq) was dissolved in 50 mL MeOH and the solution was kept in dark. An aqueous solution of NaOH (1.387 g, 34.688 mmol, 5.0 eq) was added into the flask under an ice bath. After N_3-GFLG-OMe was exhausted, MeOH was removed by rotary evaporation and 1 N HCl was added to adjust pH to 1. Finally, EA was added to extract the product, and the EA phase was washed with NaCl aq. (satd) and dried with Na_2SO_4. After removal of EA by rotary evaporation, the solid was washed with Et_2O and dried in vacuum, 2.980 g of a white solid was obtained at a yield of 98%. ^{1}H NMR (400 MHz, DMSO-d_6) δ 12.59 (s, 1H), 8.77 (t, J = 5.9 Hz, 1H), 8.13 – 8.06 (m, 3H), 7.92 – 7.87 (m, 2H), 7.24 – 7.16 (m, 7H), 4.55 (dt, J = 8.8, 4.5 Hz, 1H), 4.34 (q, J = 7.8 Hz, 1H), 3.94 – 3.85 (m, 1H), 3.80 – 3.68 (m, 3H), 3.04 (dd, J = 13.8, 4.2 Hz, 1H), 2.80 (dd, J = 13.8, 9.3 Hz, 1H), 1.66 – 1.56 (m, 1H), 1.49 (t, J = 7.3 Hz, 2H), 0.86 (dd, J = 16.4, 6.5 Hz, 6H).

1.2.2 Synthesis of N_3-GFLG-PTX

N_3-GFLG-OH (50 mg, 0.093 mmol, 1.0 eq), DCC (38 mg, 0.186 mmol, 2.0 eq), and DMAP (1 mg, 0.009 mmol, 0.1 eq) were placed into a 10 mL flask and the flask was

kept in dark. After three components were dissolved in DCM, PTX (119 mg, 0.139 mmol, 1.5 eq) was added into the flask and the mixture proceeded at room temperature for 12 h. Finally, the reaction solution was filtered to remove DCU, and the residue was purified with column chromatography with a mobile phase of DCM/MeOH (20/1, v/v). 0.118 g of a white solid was obtained at a yield of 92%. ^{1}H NMR (400 MHz, DMSO-d_6) δ 9.25 (d, J = 8.3 Hz, 1H), 8.75 (t, J = 5.9 Hz, 1H), 8.29 (t, J = 5.9 Hz, 1H), 8.08 (t, J = 8.3 Hz, 2H), 8.00 – 7.96 (m, 2H), 7.91 – 7.85 (m, 4H), 7.76 – 7.71 (m, 1H), 7.67 (t, J = 7.3 Hz, 2H), 7.58 – 7.53 (m, 1H), 7.52 – 7.41 (m, 6H), 7.22 – 7.14 (m, 8H), 6.29 (s, 1H), 5.84 (t, J = 9.1 Hz, 1H), 5.51 (t, J = 8.6 Hz, 1H), 5.40 (t, J = 8.2 Hz, 2H), 4.93 – 4.84 (m, 2H), 4.61 (s, 1H), 4.59 – 4.51 (m, 1H), 4.34 (q, J = 7.7 Hz, 1H), 4.13 – 4.07 (m, 1H), 4.01 – 3.86 (m, 5H), 3.75 (dd, J = 16.3, 5.8 Hz, 1H), 3.57 (d, J = 7.2 Hz, 1H), 3.03 (dd, J = 13.9, 4.3 Hz, 1H), 2.80 (dd, J = 13.8, 9.2 Hz, 1H), 2.37 – 2.28 (m, 1H), 2.21 (s, 3H), 2.10 (s, 3H), 1.79 (s, 3H), 1.77 – 1.72 (m, 1H), 1.67 – 1.58 (m, 2H), 1.52 – 1.42 (m, 6H), 1.01 (d, J = 10.2 Hz, 6H), 0.83 (dd, J = 15.4, 6.5 Hz, 6H).

1.2.3 Synthesis of Fmoc-PEG$_4$-Alkynyl

Fmoc-PEG$_4$-COOH (0.370 g, 0.758 mmol, 1.0 eq), HBTU (0.431 g, 1.137 mmol, 1.5 eq), and HOBt (0.154 g, 1.137 mmol, 1.5 eq) were placed into a flask. 5 mL DMF was added under an ice bath. After they were dissolved in DMF, 0.376 mL DIPEA (0.294 g, 2.274 mmol, 3.0 eq) was added into the flask. Finally, a DMF (5 mL) solution of propargulamine (0.050 g, 0.910 mmol, 1.2 eq) was added into the flask slowly. The reaction proceeded at room temperature overnight. After the reaction was finished, 100 mL EA and H_2O was added to separate the product through a separating funnel. The EA phase was washed with $NaHCO_3$ aq. (satd), 1 N HCl, and NaCl aq. (satd) sequentially. The organic solvent was removed by rotary evaporation after drying with Na_2SO_4. The residue was purified via column chromatography with a mobile phase of DCM/MeOH (50/1, v/v), and 0.389 g of an oil was obtained at a yield of 98%. ^{1}H NMR (400 MHz, DMSO-d_6) δ 8.28 (t, J = 5.5 Hz, 1H), 7.89 (d, J = 7.5 Hz, 2H), 7.68 (s, 2H), 7.42 (t, J = 7.1 Hz, 2H), 7.36 – 7.29 (m, 3H), 4.30 (d, J =

6.9 Hz, 2H), 4.21 (t, J = 6.9 Hz, 1H), 3.85 (dd, J = 5.5, 2.5 Hz, 2H), 3.58 (t, J = 6.4 Hz, 2H), 3.52 – 3.45 (m, 12H), 3.41 (t, J = 6.0 Hz, 2H), 3.18 – 3.10 (m, 2H), 3.098 (t, J = 2.6 Hz, 1H), 2.32 (t, J = 6.4 Hz, 2H). ^{13}C NMR (100 MHz, DMSO-d_6) δ 170.3, 156.7, 144.4, 141.2, 128.1, 127.5, 125.7, 120.6, 81.6, 73.4, 70.2, 70.2, 70.1, 70.0, 70.0, 69.6, 67.1, 65.8, 55.4, 47.2, 36.3, 28.2.

1.2.4 Synthesis of CTA-PEG$_4$-Alkynyl

Fmoc-PEG$_4$-Alkynyl (0.380 g, 0.725 mmol, 1.0 eq) was added into a 10 mL flask, and 4 mL DMF was then added. After Fmoc-PEG$_4$-Alkynyl was dissolved in DMF, 1 mL piperidine was added into the flask under an ice bath. The reaction was monitored by TLC. 50 mL H_2O and EA were added to extract the product after Fmoc-PEG$_4$-Alkynyl was exhausted, and the EA phase was washed with H_2O and NaCl aq. (satd) sequentially. Finally, the EA solution was dried with Na_2SO_4 and EA was removed by rotary evaporation. The residue was purified via column chromatography with a mobile phase of DCM/MeOH (20/1, v/v), and 0.218 g of an oil was obtained at a yield of 99%.

CTA (0.256 g, 0.917 mmol, 1.3 eq), HATU (0.523 g, 1.375 mmol, 1.5 eq) and HOAt (0.187 g, 1.375 mmol, 1.5 eq) were placed into a flask. 10 mL DCM and DIPEA (0.178 g, 1.375 mmol, 1.5 eq) was added sequentially. Finally, a DCM solution (5 mL) of NH_2-PEG$_4$-Alkynyl (0.218 g, 0.721 mmol, 1.0 eq) was added into the flask. After CTA was exhausted via monitoring by TLC, 50 mL DCM and H_2O was added. The DCM phase was washed with $NaHCO_3$ aq. (satd), 1 N HCl, and NaCl aq. (satd) sequentially. DCM was removed by rotary evaporation after drying with Na_2SO_4. The residue was purified via column chromatography with a mobile phase of DCM/MeOH (50/1-20/1, v/v), and 0.300 g of a red oil was obtained at a yield of 73.8%. ^{1}H NMR (400 MHz, DMSO-d_6) δ 8.27 (t, J = 5.2 Hz, 1H), 8.10 (t, J = 5.5 Hz, 1H), 7.94 – 7.88 (m, 2H), 7.69 (t, J = 7.4 Hz, 1H), 7.51 (t, J = 7.9 Hz, 2H), 3.85 (dd, J = 5.5, 2.5 Hz, 2H), 3.58 (t, J = 6.4 Hz, 2H), 3.51 – 3.45 (m, 12H), 3.41 (t, J = 5.8 Hz, 2H), 3.21 (q, J = 5.7 Hz, 2H), 3.09 (t, J = 2.5 Hz, 1H), 2.48 – 2.35 (m, 4H), 2.32 (t, J = 6.4 Hz, 2H), 1.91 (s, 3H). ^{13}C NMR (100 MHz, DMSO-d_6) δ 224.3, 170.6, 170.3,

144.6, 134.1, 129.5, 126.9, 119.2, 81.6, 73.4, 70.3, 70.2, 70.1, 70.0, 69.5, 67.1, 46.8, 39.2, 36.3, 33.8, 31.0, 28.2, 23.6.

1.2.5 Synthesis of the POEGMA-Alkynyl polymer

Reversible addition fragment chain transfer (RAFT) polymerization was performed to synthesize the POEGMA-Alkynyl polymer. Briefly, 0.026 g ACVA was dissolved in 49.6 mL DMSO, and the solution was mixed with 5.5 mL H_2O at 0 ℃. 26 mg CTA-PEG$_4$-Alkynyl and 1.808 g OEGMA (Mw: 500 Da) were simultaneously placed into a flask and the flask was sealed. 5.51 mL of an ACVA solution was added via a syringe under an argon atmosphere. After bubbling with argon for 50 min, the flask was transferred to an oil bath at 71 ℃ in dark and the reaction lasted for 20 h. The reaction solution was purified by dialyzing with deionized water (MWCO: 8 kDa) for 48 h, and then filtered with a 0.45 μm drainage filter. After drying with a lyophilizer, 1.803 g of a pink oil was obtained at a yield of 95%.

1.2.6 Synthesis of the POEGMA-GFLG-PTX (PGP) polymer

The POEGMA-Alkynyl polymer (0.958 g) and N_3-GFLG-PTX (84 mg) were placed into a flask and they were dissolved in 15 mL DMF. Under protection with nitrogen, cuprous iodide (CuI) (0.029 g) was added to the mixture and the flask was stirred at 50 ℃ in dark. After 36 h, the reaction solution was purified by dialyzing with deionized water (MWCO: 8 kDa) for 48 h, and dried with a lyophilizer. The crude product was dissolved in DMSO and purified by dialyzing against DMSO to remove excess N_3-GFLG-PTX. Finally, the dialysis bag was dialyzed against deionized water to remove DMSO. After filtering with a 0.45 μm drainage filter, the residue was dried with a lyophilizer. 0.800 g of a pink oil was obtained at a yield of 80%.

1.3 GPC analysis of polymers

Gel Permeation Chromatography (GPC) was used to obtain the average molecular weight (M_w) and the average number molecular weight (M_n) of polymers in a GPC column of Shodex Asahipak GF-510 HQ (7.5 mm ID × 300 mm L). A mixture of a 0.2 M lithium chloride solution and DMF (LiCl/ DMF = 35/65, v/v) was used as a

mobile phase at a 0.5 mL min^{-1} flow rate and an injection volume of 50 μL was applied to a 2 mg mL^{-1} sample solution. M_w, M_n, and dispersity ($Đ$) were estimated using polyethylene glycol (PEG) at different molecular weights as standards.

1.4 The drug loading content (DLC) of PTX in PGP

The content of PTX in PGP was measure by HPLC. First, different concentrations (6.25, 3.12, 1.56, 0.78, 0.39 μg mL^{-1}) of PTX was analyzed through HPLC in a Shim-pack GLST column (C18, 5 μm, 250 × 4.6 mm I.D.) with operational conditions including ACN and H_2O as a mobile phase; a detection wavelength of 227 nm by a UV-detector; and a flow rate at 1 mL min^{-1}. Gradient elution was applied to elute the analyte. The initial mobile phase was 40% (v/v) ACN and 60% (v/v) H_2O for 5 min, and the percentage of ACN was linearly increased to 70% (v/v) over next 25 min. Before the injection of a next sample, the initial mobile phase was used to equilibrate the column. The standard curve of PTX was established from peak areas at a retention time of 23.9 min with a series of concentrations.

PGP (2 mg mL^{-1}) was incubated with papain (1.5 mg mL^{-1}) in PBS (pH = 5.4) in a 37 ℃ shaker at a speed of 220 rpm. 50 μL of the solution was withdrawn and diluted in 200 μL of MeOH at 0, 1, 2, 4, 8, 12, 24, and 48 h. After filtering with a 0.22 μm organic filter membrane, the solution was analyszed by HPLC, and the DLC of PTX in PGP was calculated according to the standard curve of PTX: DLC (%) = (the weight of PTX in PGP/the weight of PGP) × 100%.

1.5 The critical assembly concentration (CAC) of PGP

The critical assembly concentration (CAC) value is often used to assess the assembly capacity of nanomaterials. The CAC value of PGP was measured via a previously reported method[2]. 31.5 mg of pyrene was dissolved in acetone to achieve a concentration of 6 ×10^{-5} M. 20 μL of the pyrene solution was added into a vial. After acetone was evaporated completely, 2 mL of a PGP solution at different concentrations (0.001~1000 μg mL^{-1}) was added into the vial, and the vial was ultrasonically treated for 0.5 h. The fluorescence spectra of the samples were acquired via a fluorescence spectrophotometer (Ex = 336 nm, Em = 360-550 nm, and slit width:

3 nm), and the ratio of the fluorescence intensity at 373 nm and 384 nm were calculated. The CAC value of PGP was determined at the inflection point in a plot of the ratio vs the PGP concentration.

1.6 Preparation and characterization of PGP and PXD101@PGP

PGP and PXD101@PGP nanoparticles were prepared using an ultrasonic method. Briefly, the PGP polymer was dissolved in deionized water and ultrasonically dispersed to prepared PGP nanoparticles. Meanwhile, the PGP polymer and PXD101 dissolved in DMSO were added dropwise to deionized water, and the mixture was ultrasonically treated. The mixture was centrifugated at 5000 rpm for 3 min to remove unpackaged PXD101 and obtain PXD101@PGP. HPLC was used to determine the PXD101 content in PXD101@PGP. A gradient program was used to elute the analyte, which was detected at 268 nm. The initial mobile phase of 25% (v/v) acetonitrile and 75 % (v/v) 25 mM NaH_2PO_4 (pH = 2.8, adjusted by acetic acid) was maintained for 5 min, and then the percentage of ACN was linearly increased to 70% (v/v) over next 15 min. The percentage of ACN was switched back to 25% (v/v) before the injection of the next sample. The flow rate was maintained at 1 mL min^{-1}. The drug encapsulation efficiency (EE%) and DLC (%) of PXD101 in PXD101@PGP were calculated via the following equations, respectively:

EE (%) = (the weight of loaded PXD101/ the weight of added PXD101) × 100%

DLC (%) = (the weight of PXD101/the weight of PXD101@PGP) × 100%

Dynamic light scattering (DLS, Brookhaven, USA) was used to measure the hydrodynamic diameter, size distribution, and zeta potential of PGP and PXD101@PGP. Transmission electron microscopy (TEM, FEI, USA) was used to observed the morphology of PGP and PXD101@PGP at 2 mg mL^{-1}.

1.7 The responsiveness of PGP and PXD101@PGP

DLS was used to evaluate the responsiveness of PGP and PXD101@PGP. PGP and PXD101@PGP were dispersed in a PBS buffer containing papain (0.75 mg mL^{-1}) at pH 5.4 or a buffer at pH 5.4 without papain, and a buffer at pH 7.4 was used as a

control. The changes in the hydrodynamic diameter of PGP and PXD101@PGP were recorded at 0, 1, 2, 4, 6, 8, and 12 h.

1.8 *In vitro* stability of PGP and PXD101@PGP

The stability of PGP and PXD101@PGP in different media (including H_2O, phosphate-buffered saline (PBS) at pH 7.4, and DMEM with 10% serum) were evaluated via DLS. Briefly, the sample solution at a concentration of 1 mg mL^{-1} was incubated in a humidified chamber (37 ℃), and the average hydrodynamic diameters of PGP and PXD101@PGP were measured at 0, 1, 2, 4, 8, 12, 24, 48, and 72 h.

1.9 *In vitro* drug release behavior of PGP and PXD101@PGP

The cumulative release of PTX or PXD101 from PGP or PXD101@PGP was evaluated under different conditions, including a PBS buffer at pH 5.4, and a PBS buffer at pH 5.4 with papain (0.75 mg mL^{-1}). Briefly, PGP or PXD101@PGP was added into different buffer solutions (2 mg mL^{-1}, 1 mL) and the mixture was transferred into a 2 kDa dialysis bag. The dialysis bags were placed in a 15 mL centrifuge tube with 10 mL of the corresponding buffer solutions (with 0.1% Tween 80). After incubation in a 37 ℃ shaker at a speed of 220 rpm, 50 μL of the buffer solution was withdrawn and diluted in 200 μL of MeOH at 0, 1, 2, 4, 8, 12, 24, and 48 h. The released amount of PTX AND PXD101 were quantified by HPLC after filtering with a 0.22 μm organic filter membrane. The parameters of HPLC for PTX was shown in **1.4** and shown in **1.6** for PXD101.

2. Supplementary Results

2.1 Supplementary Schemes

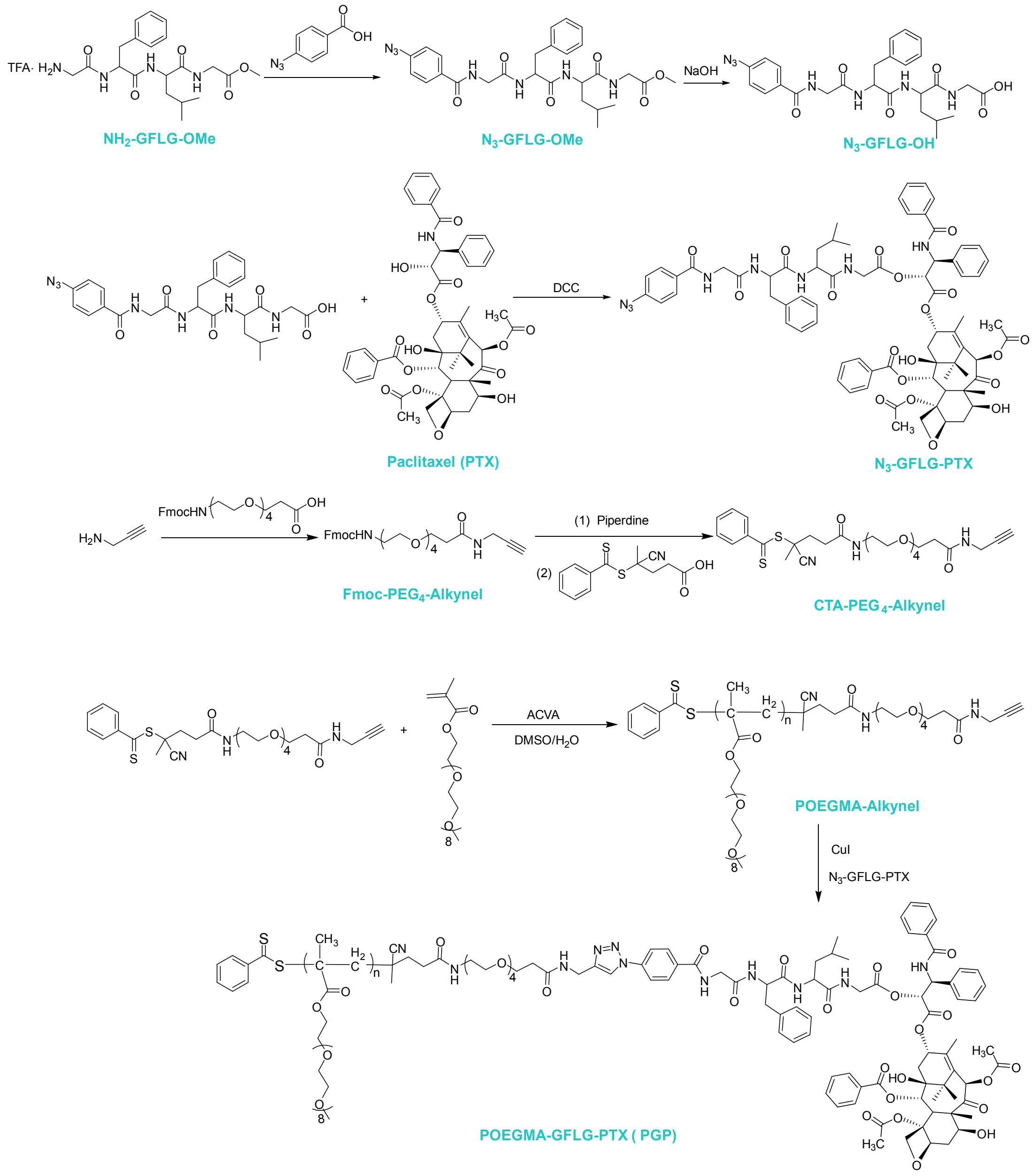

Scheme 1. Synthesis routes of POEGMA-GFLG-PTX (PGP).

2.2 Supplementary Figures

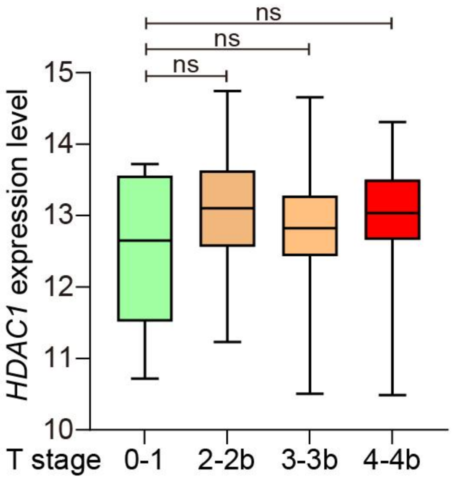

Figure S1. Relative HDAC1 mRNA expression levels across pathological T stages from a TCGA-BLCA dataset.

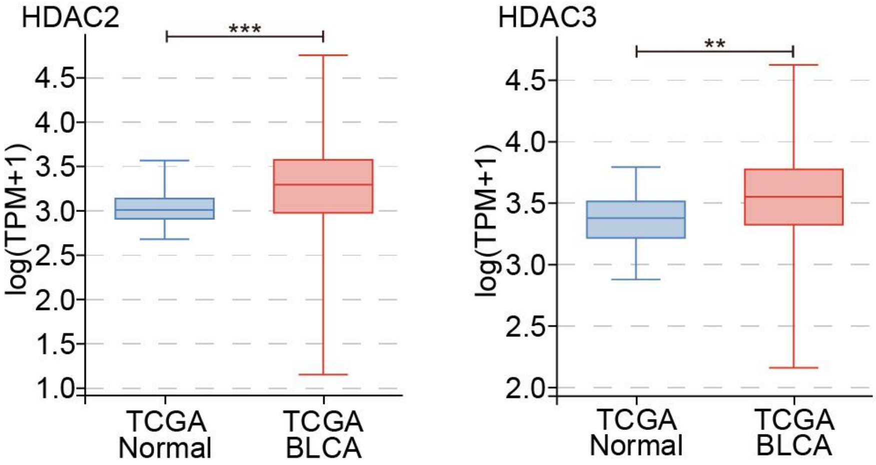

Figure S2. Relative mRNA expression levels of HDAC2 and HDAC3 from a TCGA-BLCA dataset (Normal vs Tumor).

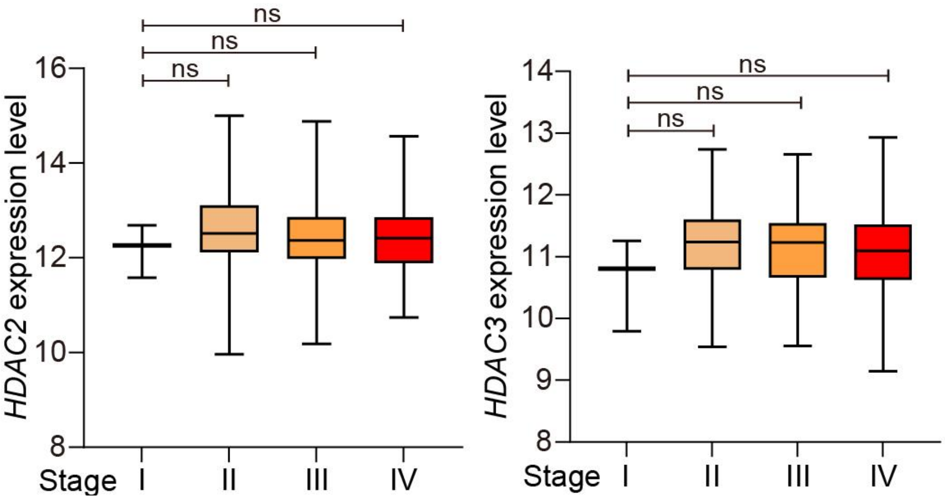

Figure S3. HDAC2 and HDAC3 expression levels stratified by tumor pathological stages.

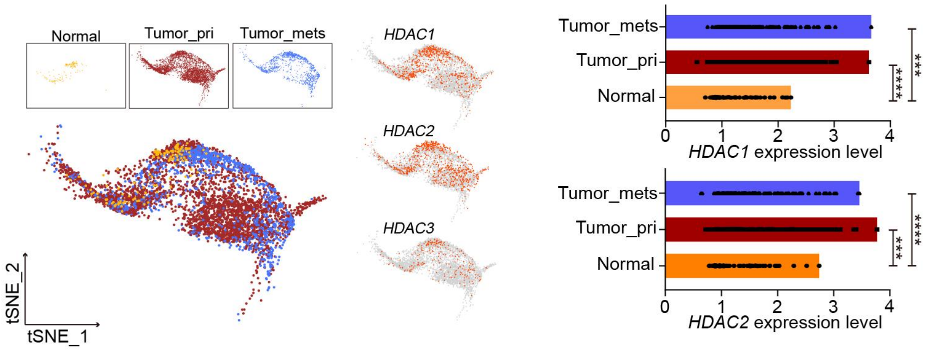

Figure S4. Relative expression levels of class I HDAC across epithelial cell types, based on single cell RNA-sequencing data.

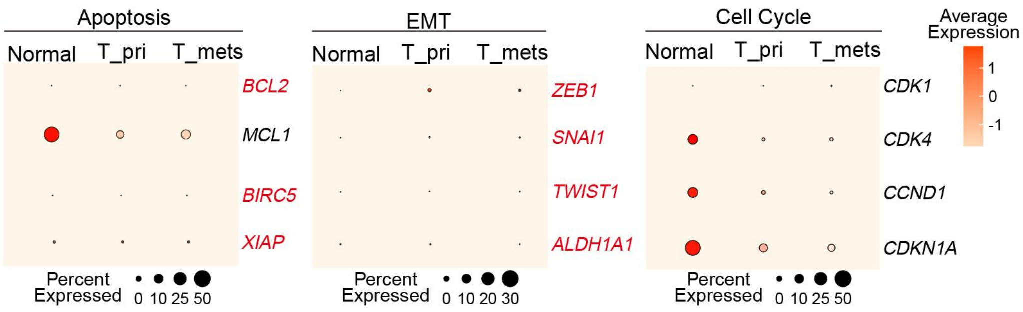

Figure S5. Enrichment scores of chemoresistance-associated pathways across normal, primary, and metastatic epithelial cells, based on single cell RNA-sequencing data.

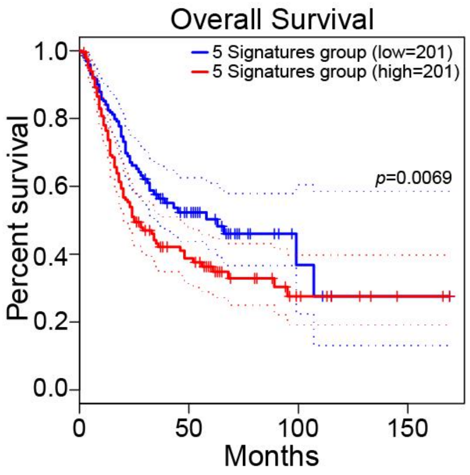

Figure S6. Kaplan–Meier analysis of overall survival stratified by expression levels of chemo-resistance-associated gene signatures.

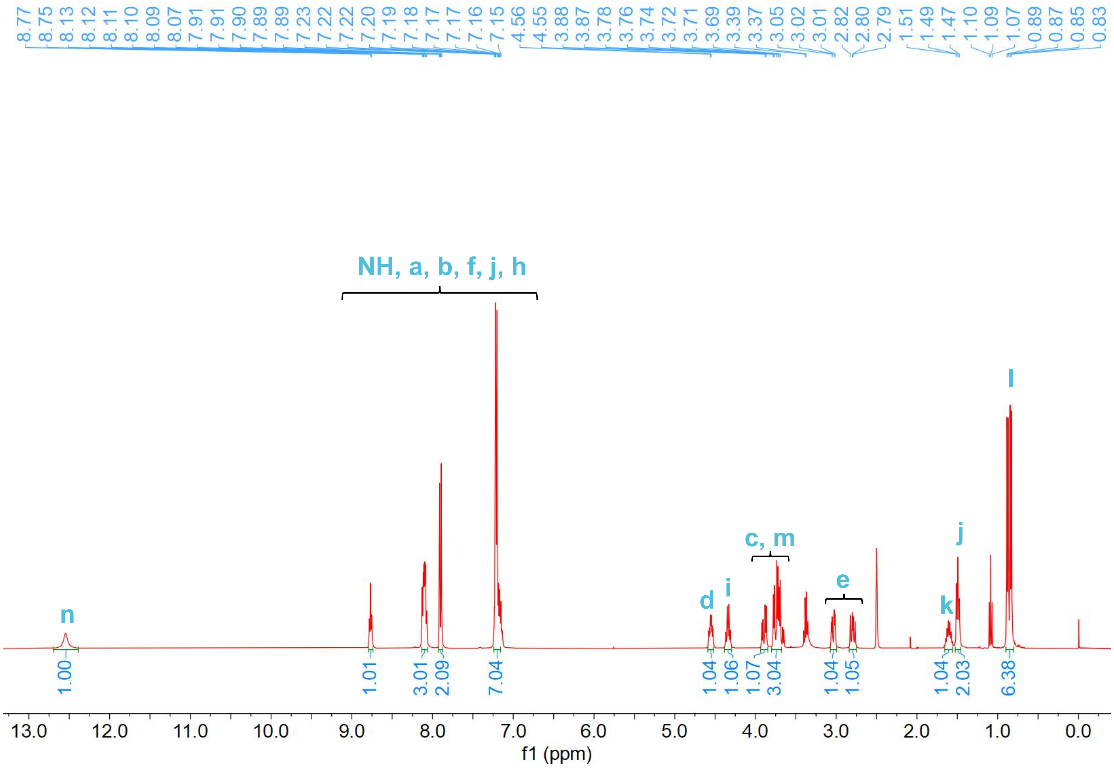

Figure S7. ^{1}H NMR spectrum of **N_3-GFLG-OH** in DMSO-d_6.

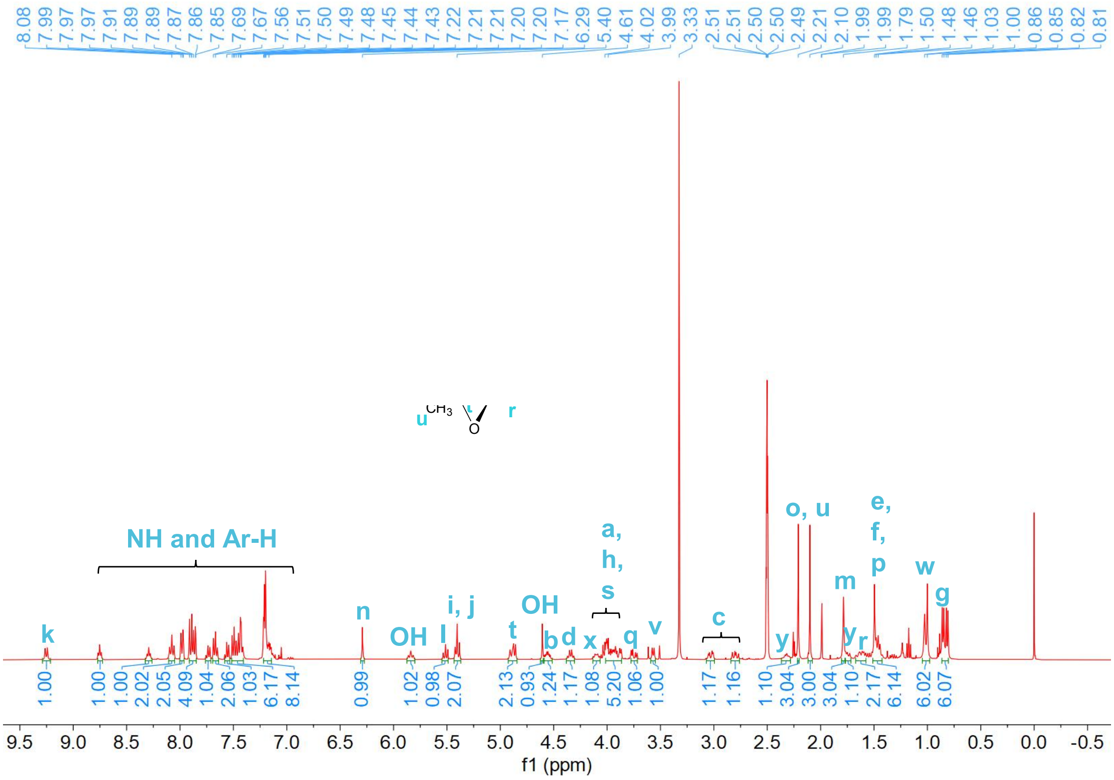

Figure S8. ^{1}H NMR spectrum of **N_3-GFLG-PTX** in DMSO-d_6.

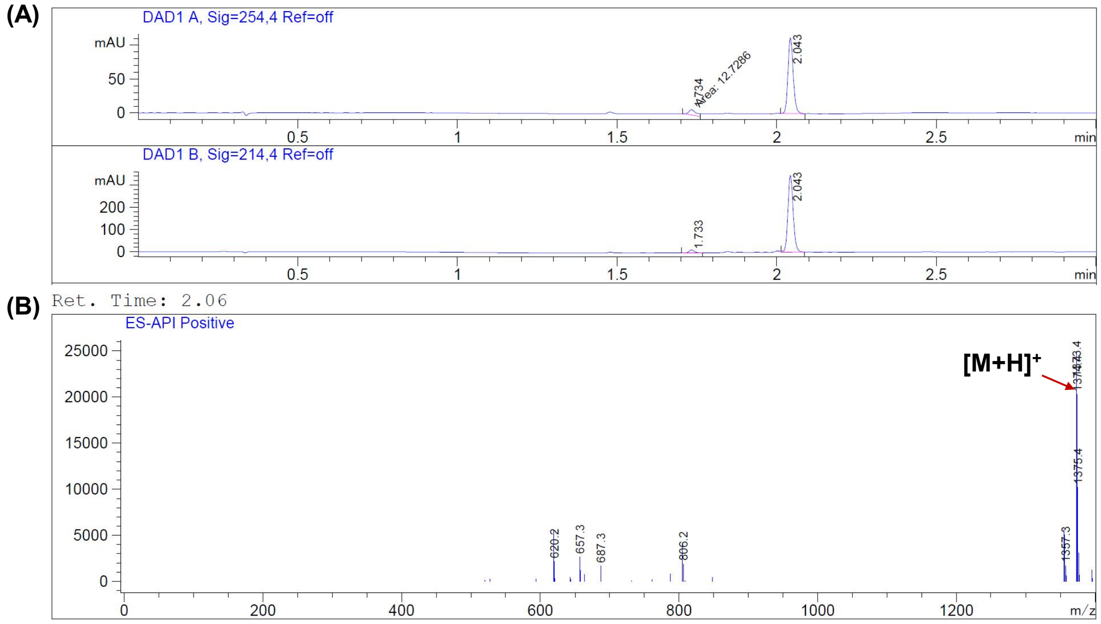

Figure S9. LC-MS spectra of **N_3-GFLG-PTX** recorded in water containing 0.01 mol L^{-1} NH_4HCO_3 and ACN. The product displayed a peak at 2.06 min (A), and a peak at 1373.4 m/z for $[M+H]^+$ (B).

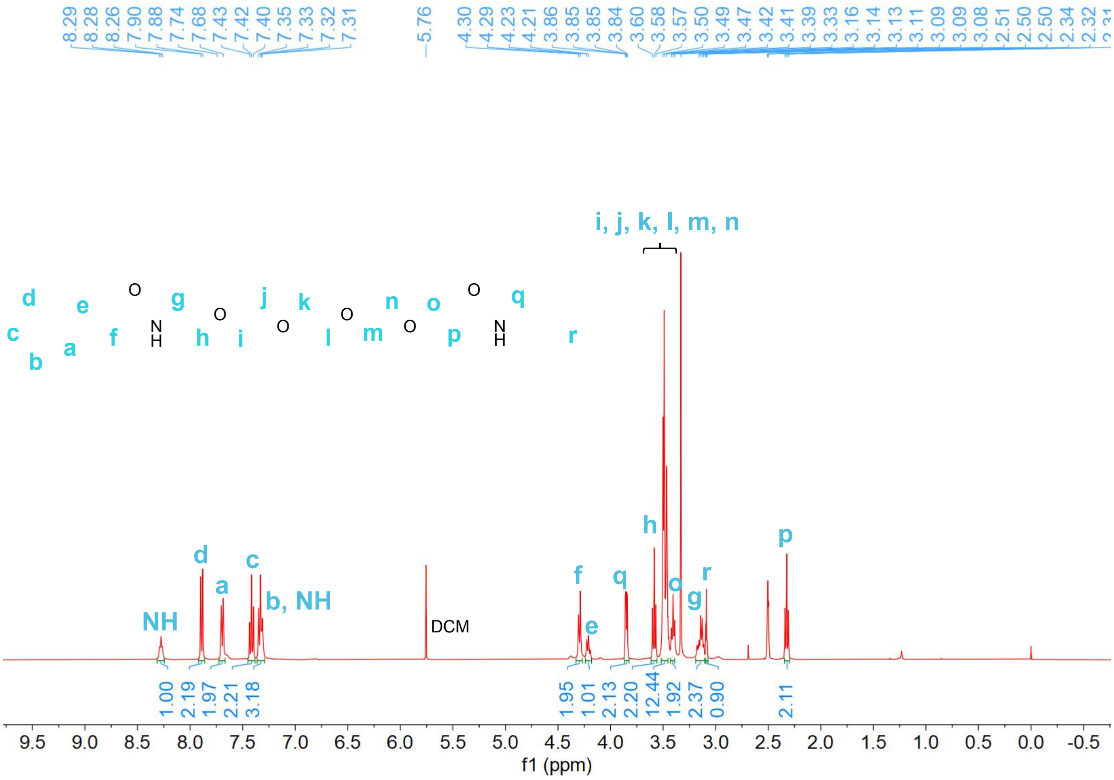

Figure S10. ^1H NMR spectrum of **Fmoc-PEG$_4$-Alkynyl** in DMSO-d_6.

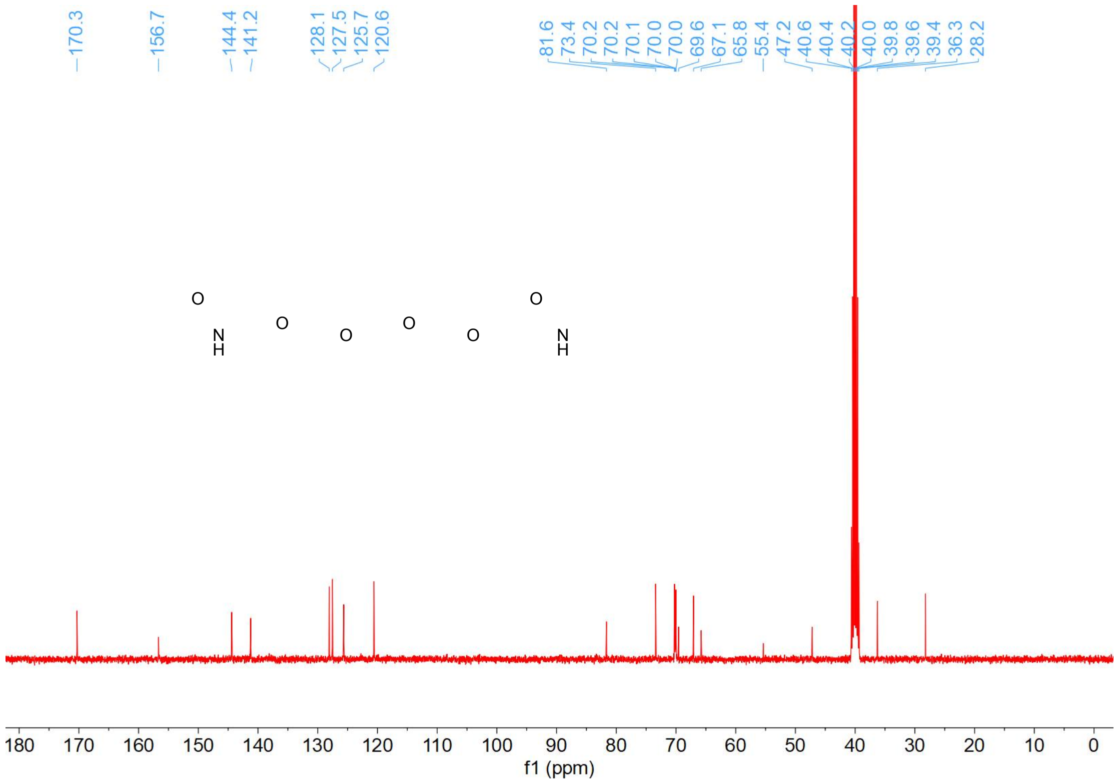

Figure S11. ^{13}C NMR spectrum of **Fmoc-PEG$_4$-Alkynyl** in DMSO-d_6**.**

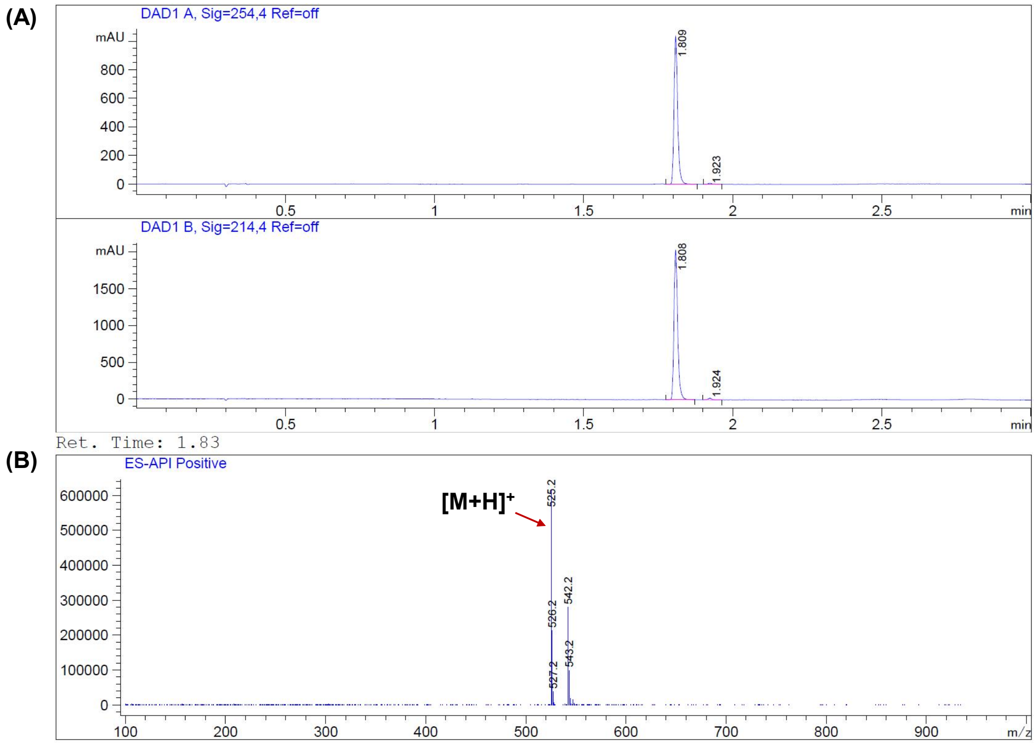

Figure S12. LC-MS spectra of **Fmoc-PEG$_4$-Alkynyl** recorded in water containing 0.01 mol L^{-1} NH_4HCO_3 and ACN. The product displayed a peak at 1.83 min (A), and a peak at 525.2 m/z for $[M+H]^+$ (B).

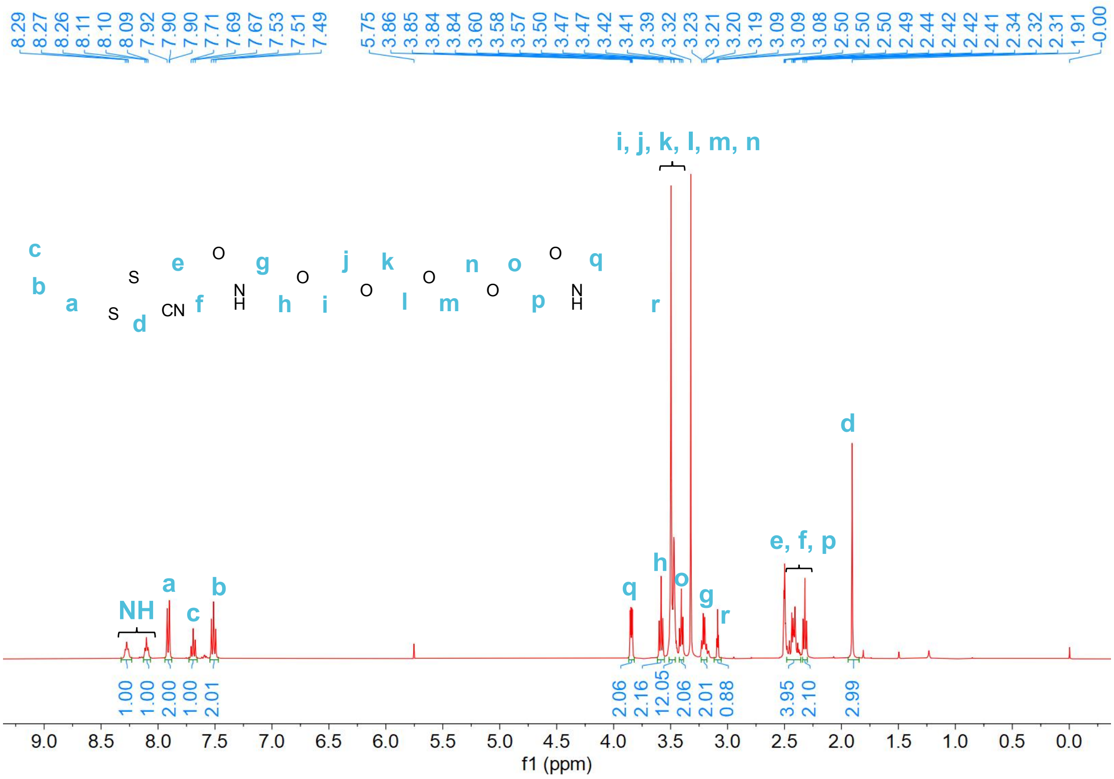

Figure S13. ^{1}H NMR spectrum of **CTA-PEG$_4$-Alkynyl** in DMSO-d_6.

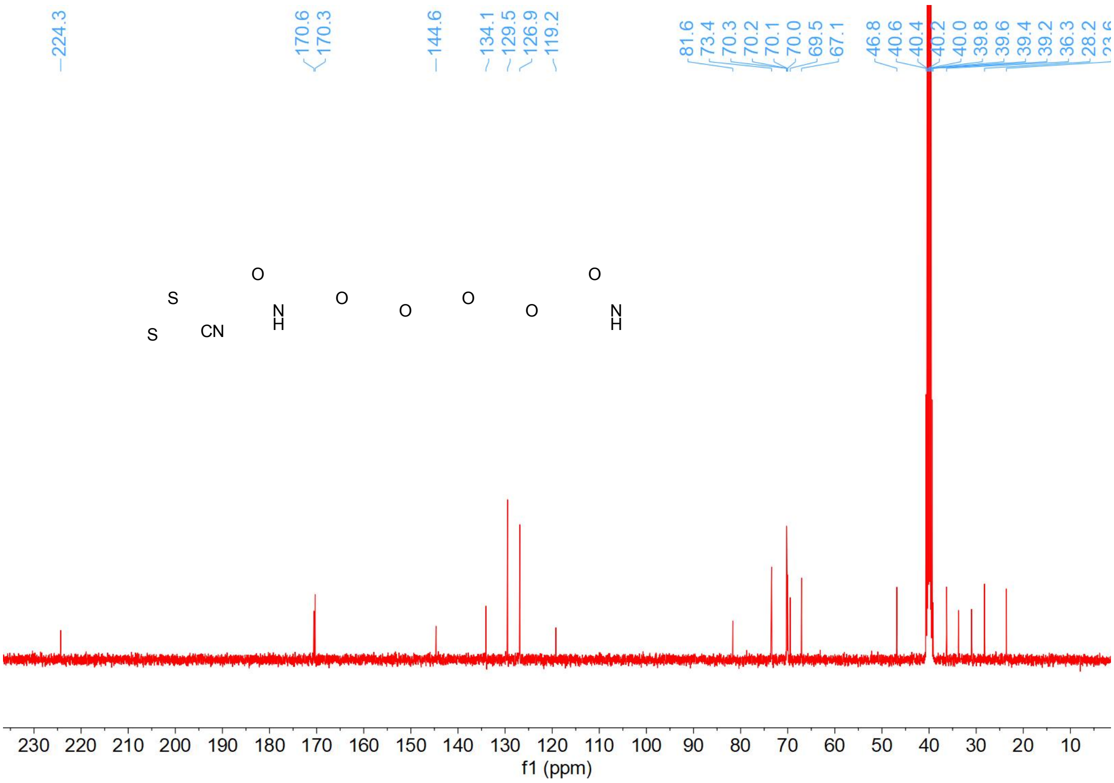

Figure S14. ^{13}C NMR spectrum of **CTA-PEG$_4$-Alkynyl** in DMSO-d_6.

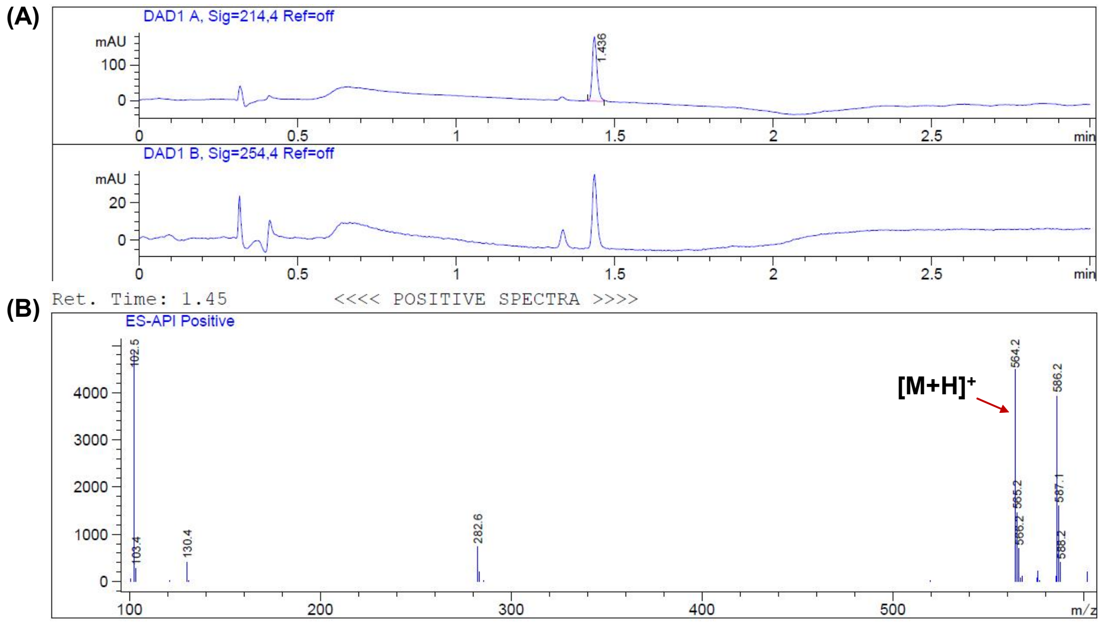

Figure S15. LC-MS spectra of **CTA-PEG$_4$-Alkynyl** recorded in water contain 0.05% TFA and ACN contain 0.05% TFA. The product displayed a peak at 1.45 min (A), and a peak at 564.2 m/z for $[M+H]^+$ (B).

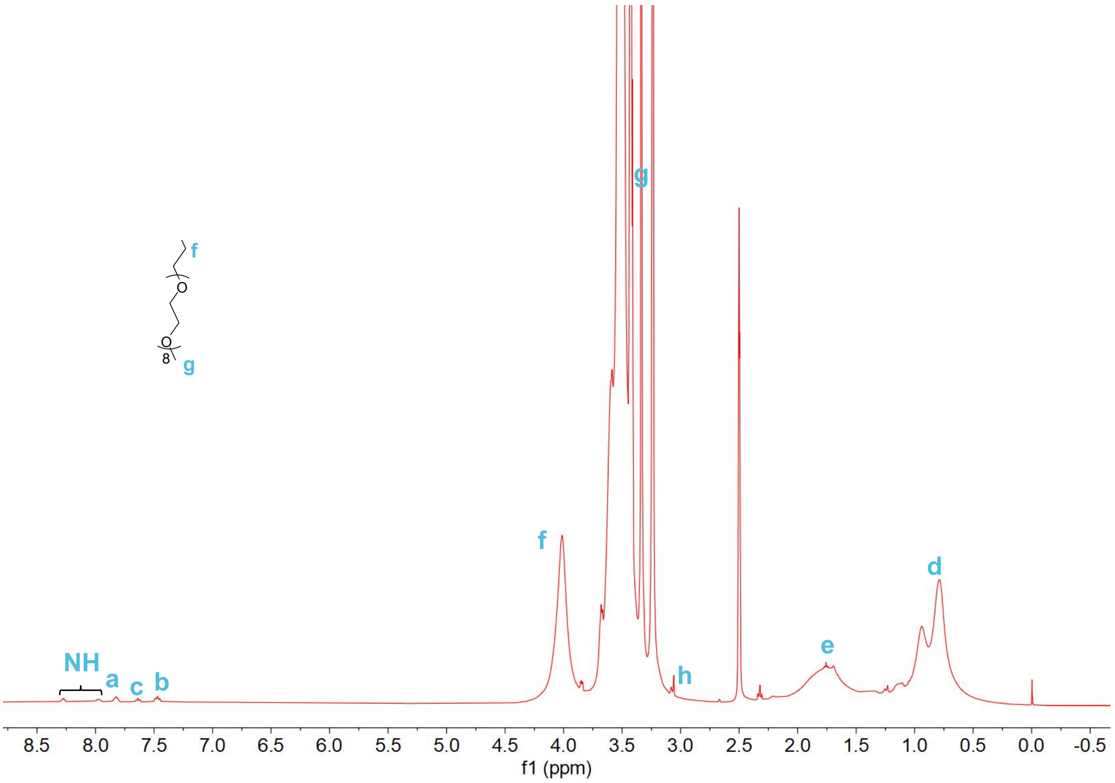

Figure S16. ^{1}H NMR spectrum of **POEGMA-Alkynyl** in DMSO-d_6.

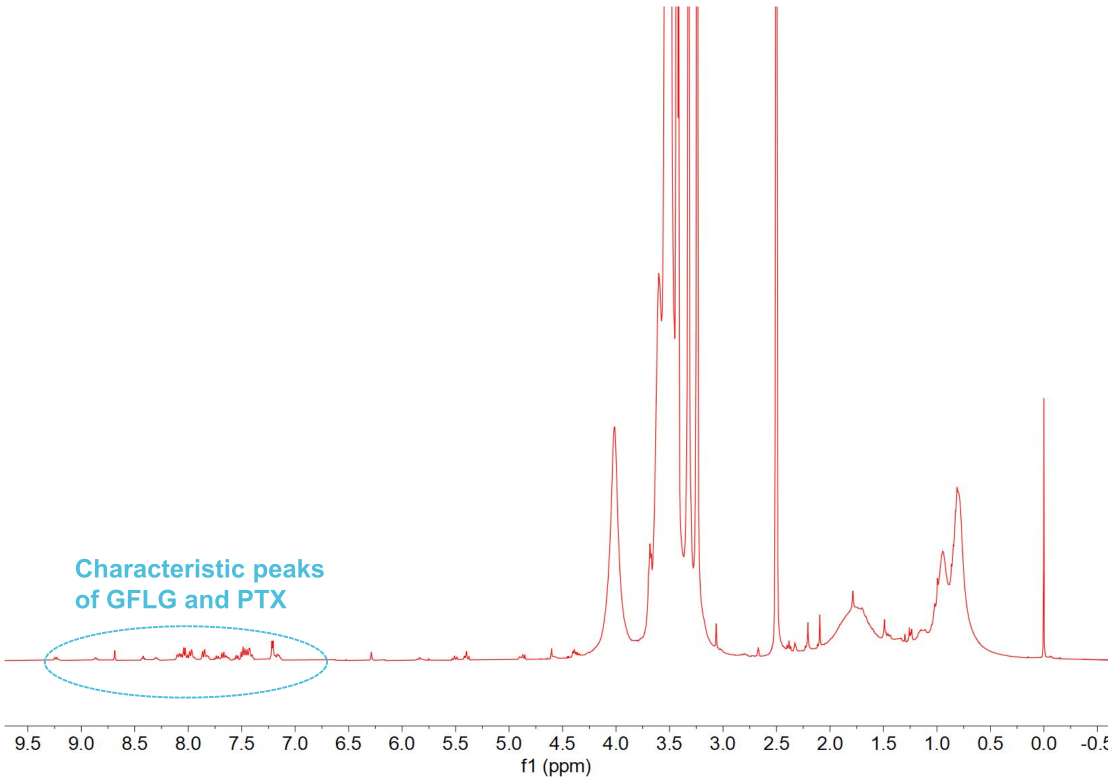

Figure S17. ^{1}H NMR spectrum of **POEGMA-GFLG-PTX** in DMSO-d_6.

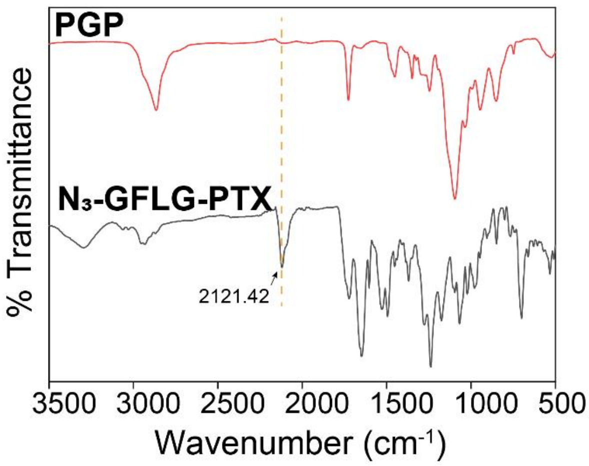

Figure S18. Fourier-transform infrared (FTIR) spectra of N_3-GFLG-PTX and PGP.

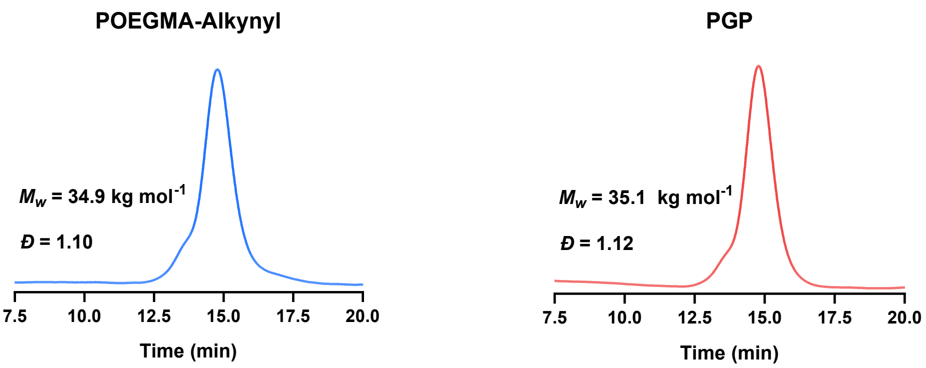

Figure S19. GPC results of POEGMA-Alkynyl and PGP.

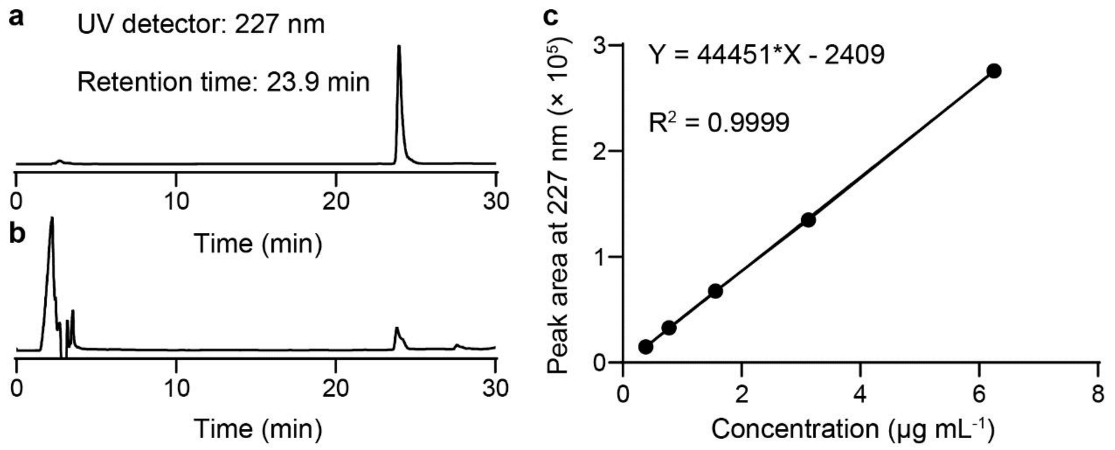

Figure S20. HPLC analysis of (a) free PTX (b) PGP incubated with papain at 37 ℃ for 48 h. (c) UV absorbance at a wavelength of 227 nm was recorded and a standard curve was established.

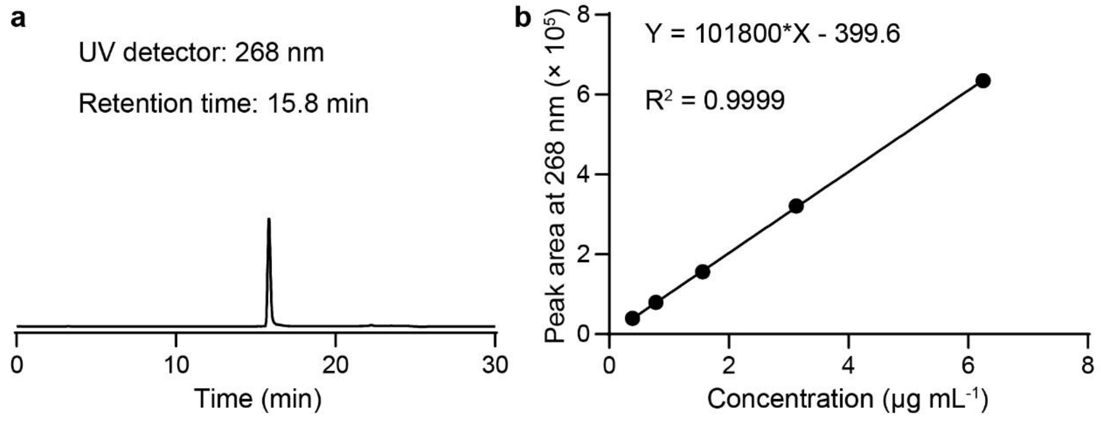

Figure S21. (a) HPLC analysis of free PXD101 at a retention time of 15.8 min. (b) The standard curve for the absorbance peak of PXD101 at 268 nm versue different PXD101concentrations.

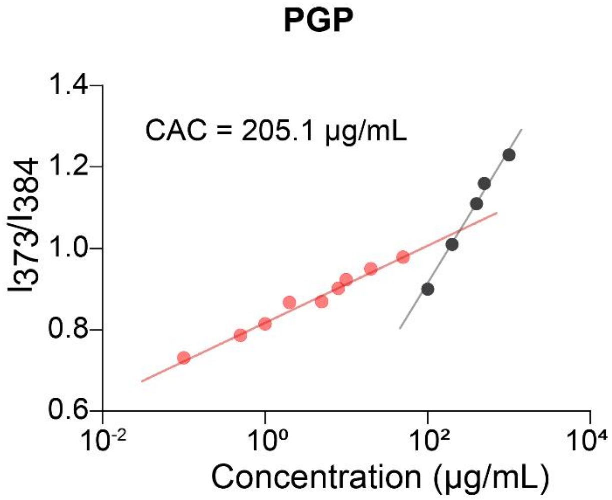

Figure S22. The critical aggregation concentration (CAC) value of PGP measured with pyrene as a probe.

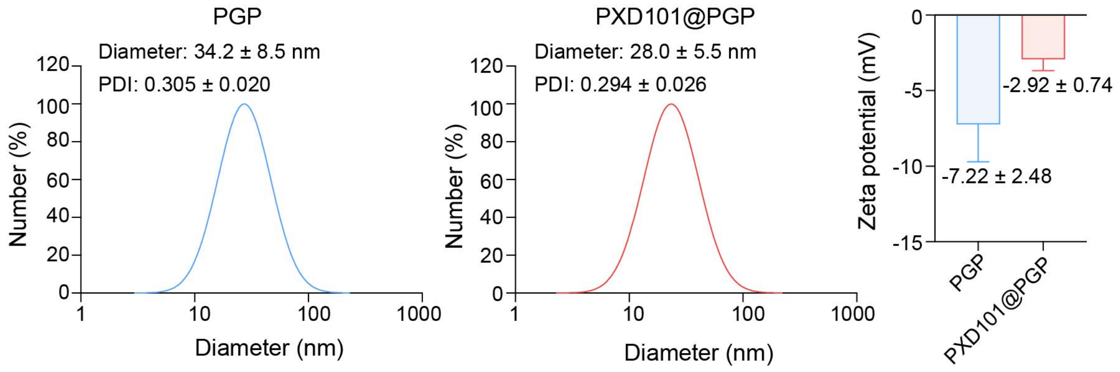

Figure S23. The hydrodynamic diameters and zeta potential of PGP and PXD101@PGP (n = 6).

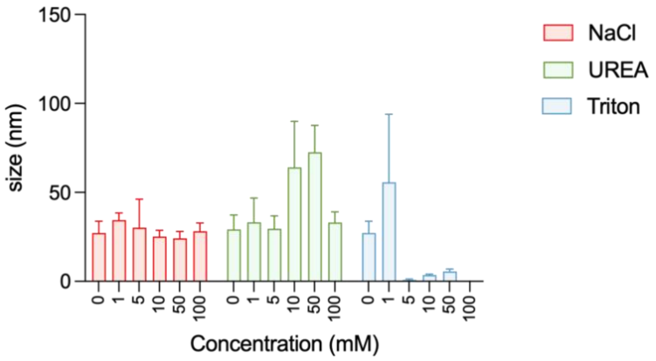

Figure S24. Formation mechanism of PXD101@PGP incubated with NaCl, Urea, or Triton at different concentrations (n = 6). Hydrogen bonds and hydrophobic interaction may predominantly drive the co-assembly of PXD101@PGP.

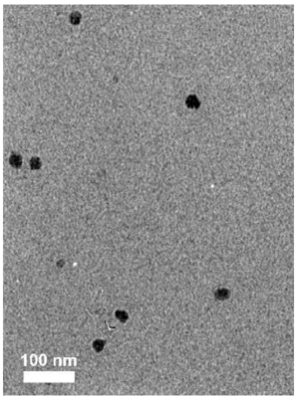

Figure S25. A typtical transmission electron microscope (TEM) image of PGP.

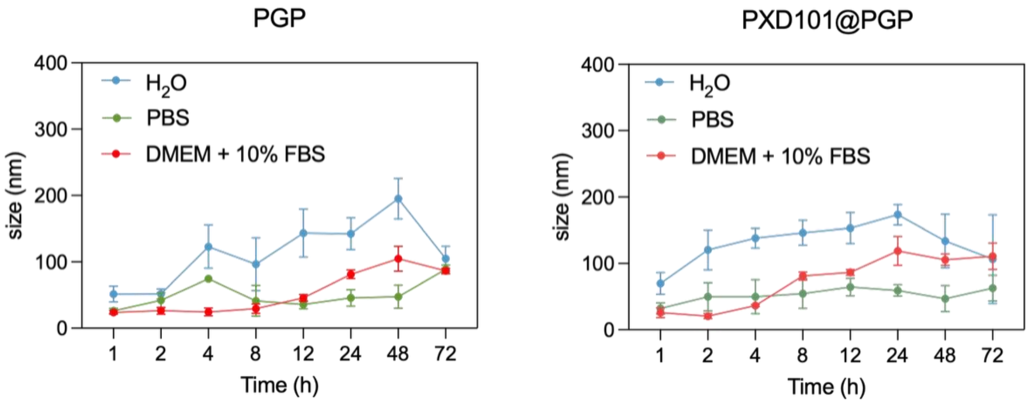

Figure S26. *In vitro stability* of PGP and PXD101@PGP in H_2O, PBS (pH = 7.4) and DMEM (10% FBS) (n = 4).

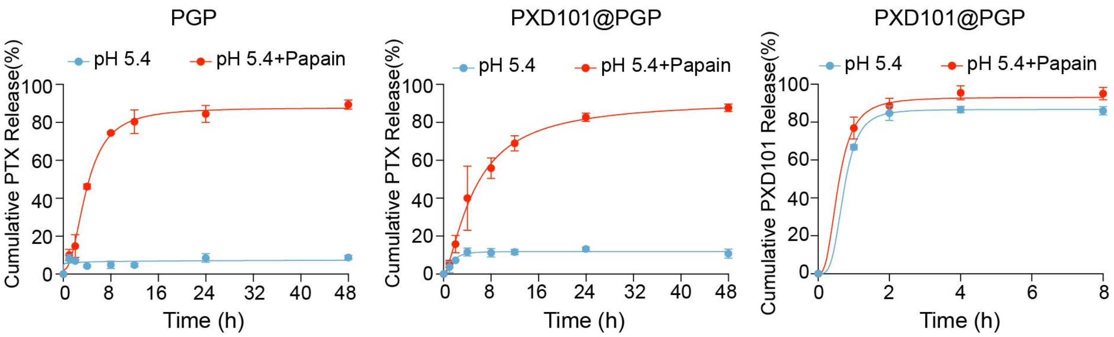

Figure S27. In vitro PTX and PXD101 release from PGP or PXD101@PGP under acidic pH (PBS, pH = 5.4) and Cathepsin B environment (0.75 mg/mL Papain in PBS, pH = 5.4). (n = 3).

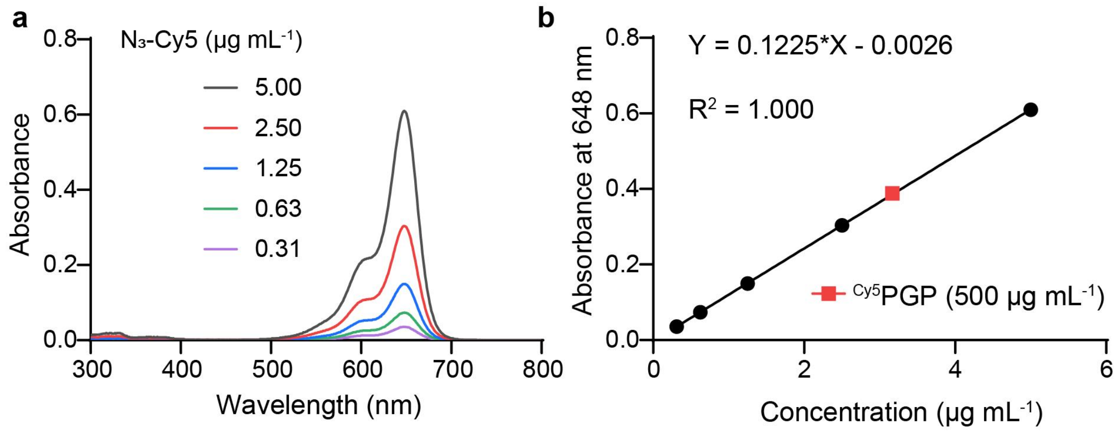

Figure S28. Determination of the Cy5 content in ^{Cy5}PGP dissolved in DMSO. (a) UV absorbance spectra of N_3-Cy5 at different concentrations. (b) The Cy5 content in ^{Cy5}PGP (500 μg mL^{-1}) based on the standard curve.

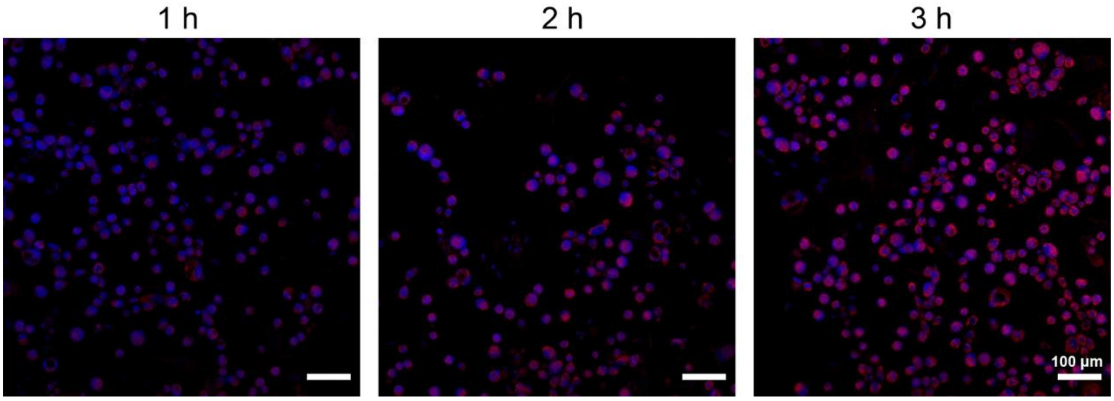

Figure S29. Representative CLSM images of primary bladder cancer cells treated with free-Cy5 at different time points (scale bar = 100 μm).

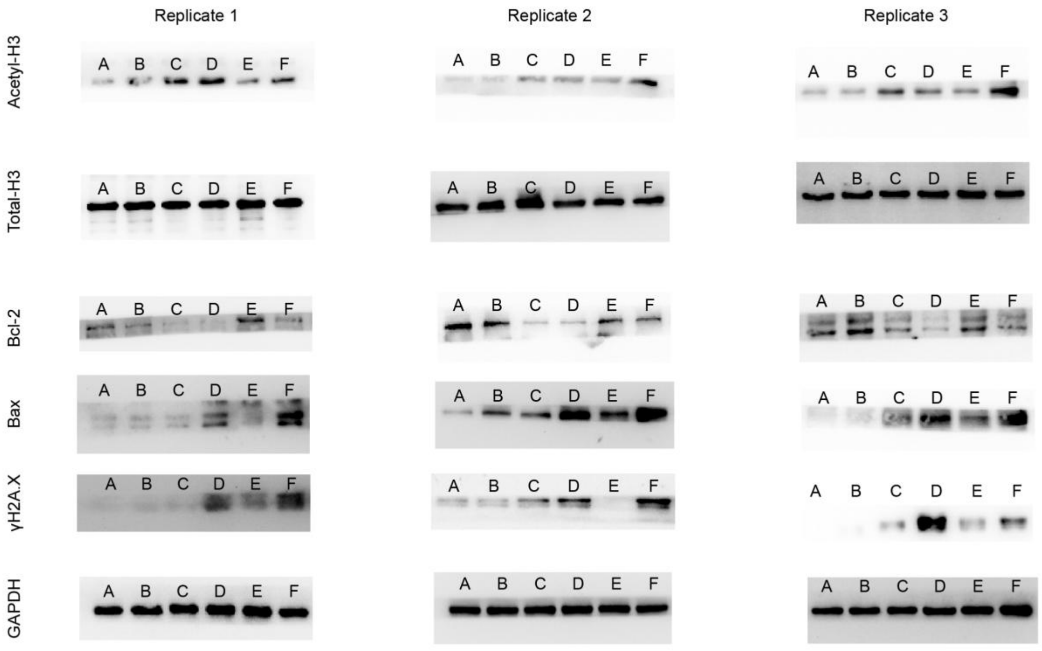

Figure S30. Western blotting results and original images of Acetyl-H3, Bcl-2, γH2A.X and Bax in primary bladder cancer cells after treatment with the control, PTX, PXD101, PTX + PXD101, PGP, or PXD101@PGP (lane A-F). Experiments were repeated three times.

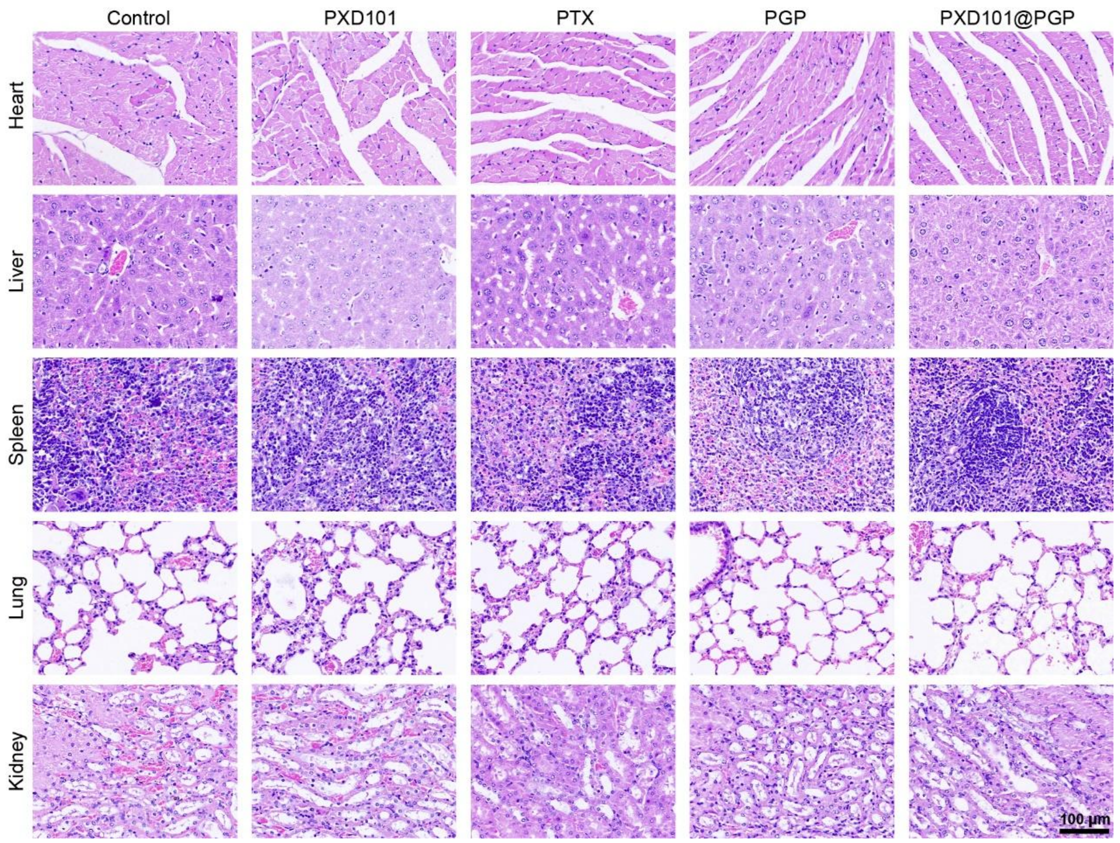

Figure S31. H&E images for major organs harvested from the chemotherapy-resistant PDX tumor model after treatment with different formulations for 13 days (scale bar: 100 μm).

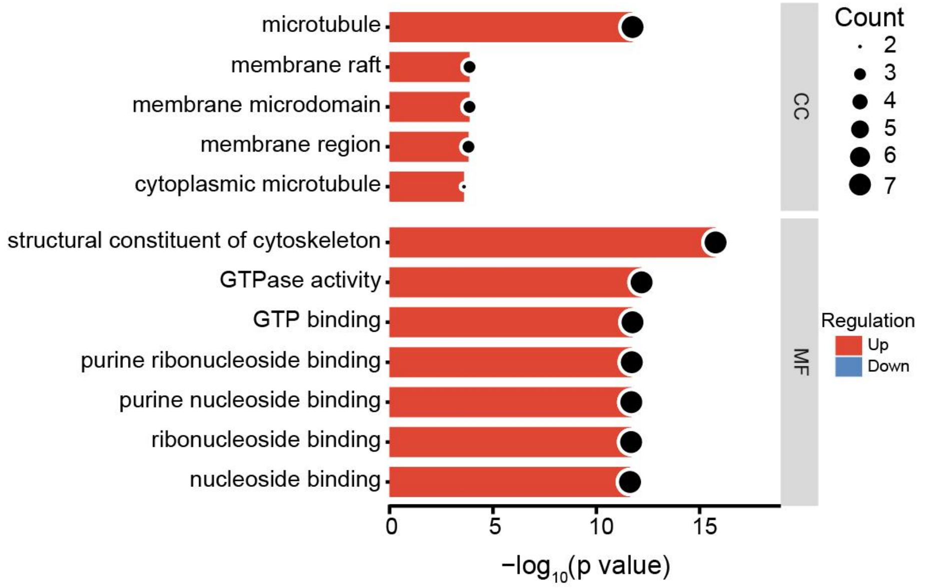

Figure S32. GO analysis of up-regulated pathways in the PXD@PGP treatment group, compared with the PXD101 treatment group.

3. Reference

[1] X. Zhi, Y. Jiang, L. Xie, Y. Li, C. J. Fang, *ACS Appl. Bio Mater.* **2019**, *2*, 5697.

[2] X. Zheng, D. Pan, M. Chen, X. Dai, H. Cai, H. Zhang, Q. Gong, Z. Gu, K. Luo, *Adv. Mater.* **2019**, *31*, 1.
